# Age-adjusted machine learning identifies facial skin microbes associated with skin quality among Korean women

**DOI:** 10.1128/msystems.00840-26

**Published:** 2026-08-20

**Authors:** Sangbeen Park, Hye-Been Kim, Hyunsoo Ahn, Geunyeong Lee, Woomin Song, Misun Kim, Eunjin Park, Byung Sun Yu, Miyang Han, Seyoung Mun, Dong-Geol Lee, Chun Ho Park, Seunghyun Kang, HyungWoo Jo, Sanguk Kim

**Affiliations:** 1 Department of Life Sciences, Pohang University of Science and Technology 123594, Pohang, Korea; 2 Research and Innovation Center, COSMAX BTI, Seongnam, Republic of Korea; 3 Department of Precision Medicine, Kyung Hee University 26723, Seoul, South Korea; 4 Graduate School of Artificial Intelligence, Pohang University of Science and Technology 34995, Pohang, Republic of Korea; 5 Division of Interdisciplinary Bioscience & Bioengineering, Pohang University of Science and Technology 34995, Pohang, Republic of Korea; 6 Department of Biomedical Sciences, College of Bio-convergence, Dankook University 477888, Cheonan, Republic of Korea; 7 Department of Microbiology, College of Science & Technology, Dankook University 34937, Cheonan, Republic of Korea; 8 Center for Bio-Medical Engineering Core Facility, Dankook University 65383, Cheonan, Republic of Korea; 9 Department of Cosmedical Materials, College of Bio-convergence, Dankook University 477888, Cheonan, Republic of Korea; 10 Research and Innovation Center, COSMAX 476617, Seongnam, Republic of Korea; The University of Hong Kong, Hong Kong, Hong Kong

**Keywords:** skin microbiome, bioinformatics, age, cosmetology, skin quality

## Abstract

Recognizing specific microbes that significantly influence skin quality is becoming an essential aspect of personalized skincare. However, conventional large-scale cohort skin microbiome studies often overlook important confounders, such as age, leading to missing meaningful microbe-skin relationships. In this study, we developed an age-adjusted machine learning (AAML) framework to identify microbial candidates associated with skin quality by determining optimal age ranges that enhance age-independent signals of skin microbes. It allowed the identification of distinct age groups that clearly explain specific skin microbial effects, as well as potential microbes showing notable age-independent links to skin quality, which were not observed in analyses across the entire age spectrum. In particular, *Corynebacterium propinquum* (*C. propinquum*) was recognized as a key species that positively impacts the middle-aged group, especially regarding skin tone. We further validated its dermatological significance using functional assays in human skin cell lines, performed a gene-level functional analysis, and suggested a potential mechanism. Our AAML method can be adapted to other microbiome analyses to precisely measure factors unaffected by age-related confounding factors.

**IMPORTANCE:**

Age is a crucial but often intractable confounder in microbiome studies, obscuring how specific microbes affect human traits. We developed an age-adjusted machine learning (AAML) framework that automatically finds age ranges where the microbiome best predicts skin quality, rather than relying on arbitrary age groups. In a Korean facial skin cohort, AAML revealed three biologically meaningful age windows and uncovered microbial effects that are invisible in whole-age analyses. AAML identified *Corynebacterium propinquum* as a previously unrecognized commensal microbe that improves skin tone in the middle-aged group, and we mechanistically linked this effect to resveratrol production. Our framework provides a general, confounder-aware strategy for discovering age-independent microbiome-host relationships.

## INTRODUCTION

With the rapid expansion of the personalized skin care industry, the skin microbiome is garnering increasing interest as a critical factor that fundamentally influences individual skin appearance (1, 2). Growing evidence indicates that commensal microorganisms influence skin characteristics, such as moisture, tone, barrier integrity, and texture (3–5). Consequently, identifying skin microbes that strongly impact skin quality has become a key goal in skin research. Recently, large-scale cohort studies have increased statistical power to identify the links between skin microbes and skin features using quantitative skin measurements and skin microbiome profiles (6–9). These efforts have also connected specific microbes to functional pathways responsible for phenotypes such as hydration and skin health tone (6).

However, statistical analyses of such cohorts often overlook the continuous confounding factors of the skin microbiome, leading to missing microbe-skin quality relationships. If analyses are conducted at full scale without accounting for factors such as age, lifestyle, geography, and environmental exposure, they often fail to identify factor-specific or opposing microbial effects on skin quality (9–21). Among these confounding factors, age exerts one of the most pervasive influences (9, 22, 23). For example, age simultaneously drives a natural decrease in both *Cutibacterium acnes* abundance and overall skin quality (24). Due to this correlation, age acts as a direct confounder artificially skewing the data to suggest that *C. acnes* universally improves skin quality simply because of its high prevalence in youth. Consequently, this confounding effect masks the microbe’s negative impacts. In reality, *C. acnes* provides beneficial effects by producing short-chain fatty acids that support barrier integrity and reduce inflammation (25–27); however, the same species contributes to acne pathogenesis in adolescents and young adults via cutaneous inflammation (27–30). This contrast arises because aging progresses nonlinearly (31–34), encapsulates other covarying factors (22, 35), and drives temporal shifts in both microbial abundance and skin physiology (36). These observations highlight the need for group-specific studies to identify hidden microbial signals within each age group, rather than relying on a single model for all ages.

A significant challenge lies in establishing appropriate age boundaries to identify microbial signals. Skin quality-microbiome association studies often struggle to capture generalizable microbial effects because they rely on arbitrary boundaries. Recent studies have divided participants into predefined age ranges to minimize age-related confounding of skin quality (24, 37–40). However, these predefined age boundaries do not fully capture the biological diversity and complexity of aging. Individuals classified into the same predefined age groups exhibited divergent biological aging profiles (37, 41). This fixed age category weakens the biological validity of age-confounding effects, ultimately limiting the detection of hidden microbial influences on skin quality. These challenges highlight the need for a computational framework optimized to emphasize the age-independent skin microbial signal.

Here, we developed an age-adjusted machine learning (AAML) framework to identify microbial candidates associated with skin quality by modeling optimal age windows that maximize age-independent signals of skin microbes. With 595 samples from the Korean facial skin microbiome data set (39), we identified three age groups that optimally elucidate the relationship between microbial composition and skin quality. Microbial candidates with high AAML contribution scores showed significant, age-independent correlations with skin quality that were not evident in analyses across the entire age spectrum. Notably, our framework identified *C. propinquum* as a beneficial species in the middle-aged group, particularly regarding skin tone. Functional assays further confirmed that *C. propinquum* suppresses melanin secretion in human skin cell lines, supporting its dermatological relevance and validating the AAML-derived microbial candidate. This optimization of age windows enhanced both microbial explanatory power and interpretability compared to conventional whole-age approaches. Overall, our findings demonstrate that AAML can effectively capture age-independent microbial signals that underline skin quality while minimizing age confounding. This framework is expected to provide a foundation for the rational development of microbiome-based, age-targeted cosmetic interventions.

## RESULTS

### The impact of age on facial microbiome and skin health

**Fig 1:**
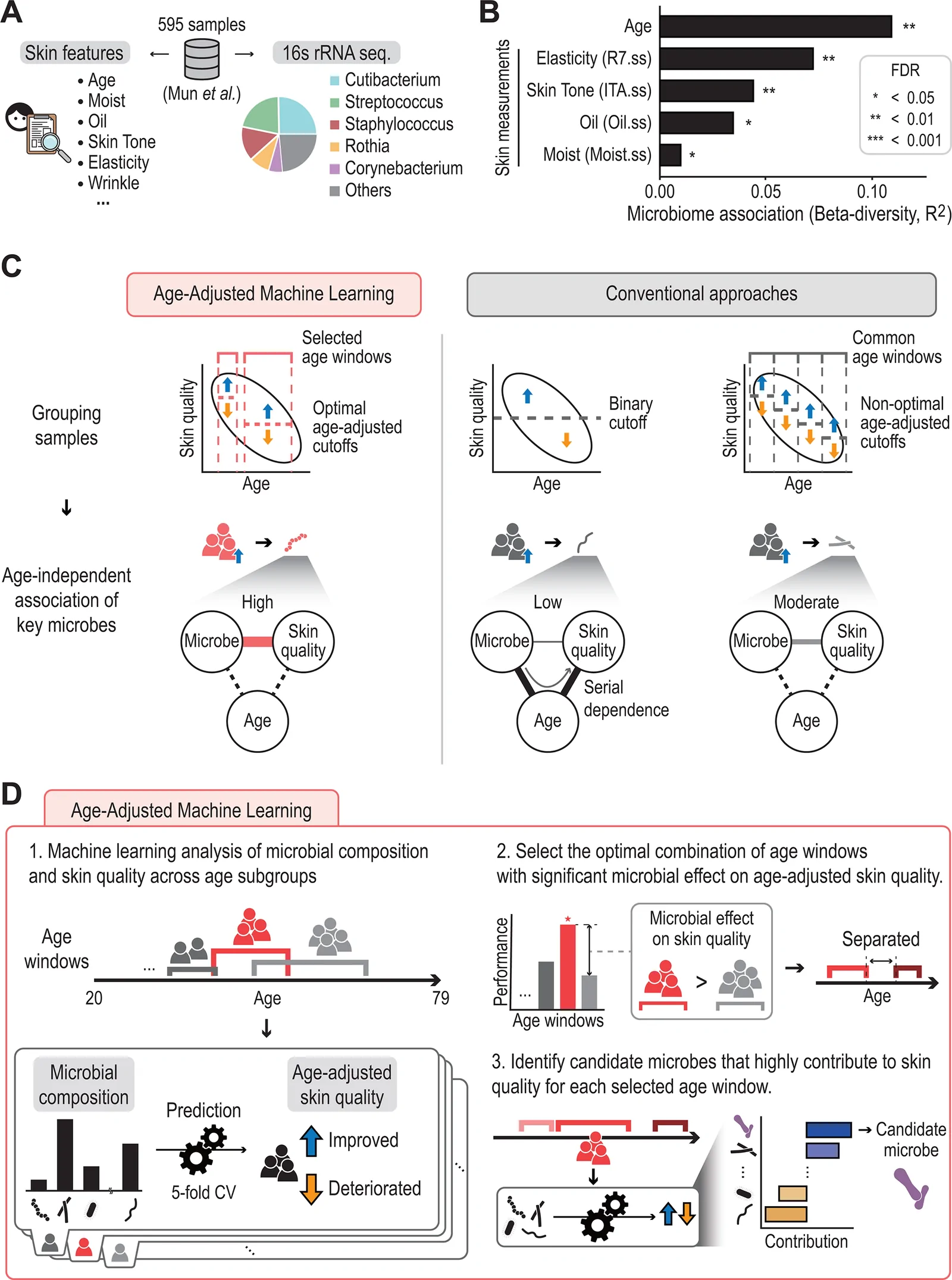
The profound effect of age on facial skin data and a framework for age-adjusted machine learning. (**A**) The curation of skin features and microbial abundance from 16S rRNA sequencing data. (**B**) Beta-diversity plot showing association with microbiome abundance and skin measurements and age, determined by Bray-Curtis dissimilarities (*, FDR < 0.05; **, FDR < 0.01; ***, FDR < 0.001; ns, not significant). (**C**) Concept of the AAML framework compared to conventional approaches. With optimal age windows and adjusted cutoffs that minimize age-related confounding, AAML amplifies the microbial signal that contributes to skin quality. (**D**) The overall process of the AAML framework consists of three steps. (1) Measuring the 5-fold CV performance of skin quality predicting ML with microbial composition for various age windows. (2) Selection of the optimal combination of age windows that maximizes microbial effect on skin quality through ML performance. (3) Identification of candidate microbes strongly associated with skin quality independently of age.

The analysis of microbial composition and skin quality showed that age is the main confounding variable. We used a Korean facial skin microbiome data set comprising 595 female samples, including both skin feature and 16S rRNA sequencing data (39) (Fig. 1A). This data set comprises a balanced age distribution and includes 50 skin-related features such as age, moisture, oil, tone, and elasticity (Fig. S2B; Table S4). Microbial abundance profiles were derived from the 16S rRNA sequencing data (Table S5; see Materials and Methods). The skin features demonstrated explanatory power concerning microbial composition (Fig. 1B; Fig. S1; see Materials and Methods). The representative skin features showed significant correlation with microbial diversity (elasticity, R2 = 7.2%, false discovery rate [FDR] = 0.002; skin tone, R2 = 4.4%, FDR = 0.002; oil, R2 = 3.4%, FDR = 0.002; moist, R2 = 1.0%, FDR = 0.038). Among the features, age emerged as the strongest factor associated with microbial variation and skin quality, showing the highest contribution to beta-diversity (R2 = 10.9%, FDR = 0.002) (Fig. 1B; Table S6). Moreover, we evaluated the age effects on skin measurement by conducting principal component analysis (PCA) using four non-redundant composite features: integrated oil (Oil.ss), integrated moisture (Moist.ss), skin tone (ITA.ss), and elasticity (R7.ss) to avoid skin measurement collinearity. Following this dimensional reduction, we stratified individuals into three distinct groups: young, middle, and old (Fig. S2A). Samples showed significant clustering along the PC1 axis (Mann-Whitney *U* test, *P* < 2.22 × 10^–16^), confirming its role as a major driver of diversity in skin quality. Together, these results reveal the profound effect of age on facial microbiome diversity and skin quality and underscore the necessity of age adjustment when identifying microbial candidates that contribute to skin quality.

For further analysis of age adjustment in the relationship between skin quality and microbial composition, we calculated a reduced-dimensional skin quality score that reflects improvements or deteriorations in skin measurements. The skin quality score was derived by integrating the four major measurements—oil, moisture, tone, and elasticity—to maximize variation (see Materials and Methods). Regarding the skin quality score and age, whole-age samples were divided into improved and deteriorated groups (Fig. S2C; see Materials and Methods). Comparing the two groups, we confirmed that positive measurements, such as cheek elasticity, were significantly higher in the improved group, whereas negative measurements, such as wrinkles, were significantly higher in the deteriorated group (Fig. S2D). Most skin measurements (41/49) showed a significant relationship, indicating that the calculated skin quality score reflected true skin quality (Fig. S2D; Table S7).

### Discovering microbial candidates using age-adjusted machine learning

We developed an AAML method that identifies microbial candidates that are strongly associated with skin quality independently of age (Fig. 1C). The primary objective of AAML is to identify microbes that exert an age-independent effect on skin quality, meaning their statistical association with skin quality is a true functional relationship rather than an artifact of chronological age confounding.

To achieve this, the AAML pipeline establishes a robust association between microbes and skin quality by selecting optimal age windows and age-adjusted thresholds (Fig. 1C and D). The pipeline first trains multiple machine learning models to predict skin quality from microbial composition for each sliding age window, ranging from short to long durations. Then, the algorithm identifies the best combination of non-overlapping age groups that maximizes the age-independent association. Finally, the AAML model identifies microbial candidates that have high predictive association to skin quality within each chosen age group. By calculating the contribution scores of microbes within these trained models, AAML pinpoints the most influential microbes based on their positive or negative impact on skin quality for a specific age group. Through this process, the framework effectively isolates true functional microbial drivers from background aging noise.

### AAML strengthened the link between microbes and skin quality by presenting the optimal age groups

**Fig 2:**
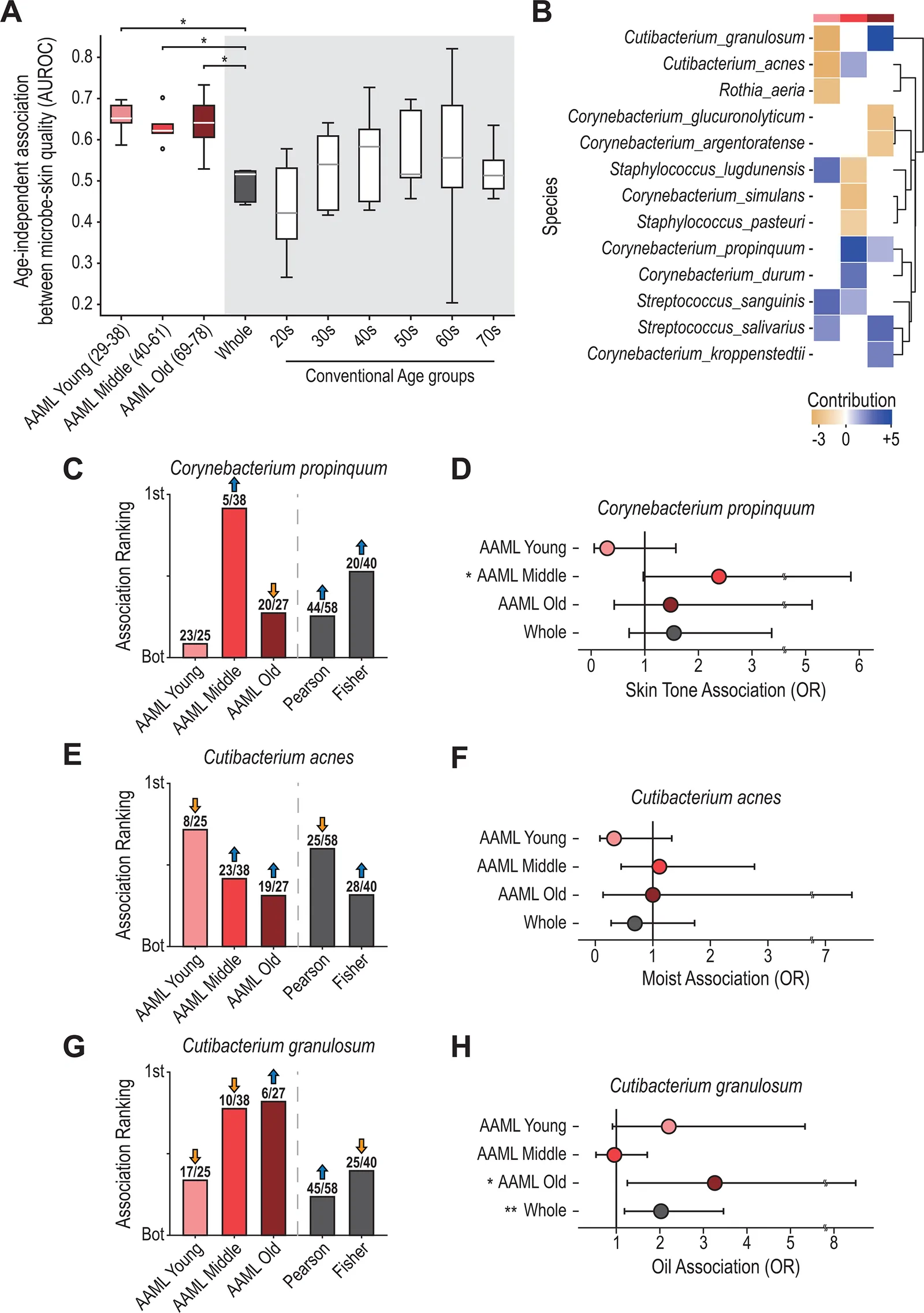
Identification of microbe candidates affecting skin quality by age group. (**A**) The comparison of performance with the samples in the AAML age group with the whole age and the conventional age group. The whole age group indicates the age 20–79 samples. The conventional age groups indicate performance using samples with a 10-year age cutoff from 20 years. Statistical significance was assessed using the Benjamini-Hochberg multiple-testing (*, FDR < 0.05; **, FDR < 0.01; ***, FDR < 0.001). (**B**) Heatmap of AAML contribution scores for microbial candidates. The contribution score is calculated as a *z*-score relative to the feature weight in the LogisticRegression ML. The microbes are clustered with hierarchical clustering methods. Blue indicates a positive contribution score, whereas yellow indicates a negative contribution score. (**C, E, and G**) Bar plot for comparing the association rank of the skin quality score between the age-adjusted sample analysis and the whole-age sample statistical analysis. The blue upper arrow indicates the improved skin quality effects, and the yellow downward arrow indicates the deteriorated skin quality effects. For the whole-age models, “Pearson” represents the association rank between continuous microbial abundance and skin quality using Pearson correlation, and “Fisher” represents the association rank between microbial presence/absence and skin quality using Fisher’s exact test. (**D, F, and H**) Forest plot for Fisher’s exact test of microbial candidates with skin measurement. (*, *P* < 0.05; **, *P* < 0.001; ***, *P* < 0.001; none, not significant).

Applying AAML to the cohort, we identified three age groups with the strongest age-independent association between microbes and skin quality. AAML identified distinct, non-overlapping groups within specific age ranges that showed significantly better predictive performance for skin quality based on microbe abundance compared to the model using the entire age spectrum (Fig. S3C; see Materials and Methods). The resulting age groups were 29–38 years for AAML-Young, 40–61 years for AAML-Middle, and 69–78 years for AAML-Old (the mean area under the receiver operating characteristic curve (AUROC) values of AAML-Young, AAML-Middle, AAML-Old, and Whole age group are as follows: 0.652, 0.631, 0.638, and 0.491, respectively), showing significantly higher association than the whole age group (FDR of AAML-Young, AAML-Middle, and AAML-Old: 1.46 × 10^–2^, 1.46 × 10^–2^, and 1.46 × 10^–2^, respectively) (Fig. 2A; Table S8). Notably, the approach using the defined age groups performed worse than the AAML model (the mean AUROC values for individuals in their 20s, 30s, 40s, 50s, 60s, and 70s are as follows: 0.431, 0.527, 0.563, 0.570, 0.550, and 0.527, respectively). This indicates that the conventional age grouping limits the explanatory power of microbial composition regarding skin quality (Fig. 2A; Table S8).

We verified that the AAML groups demonstrate significantly higher age-independent associations without increasing the risk of overfitting. We evaluated the risk of *post hoc* optimization bias by analyzing the relationship between the microbe-skin quality association and the proportion of overlap with the AAML groups (see Materials Methods). If the AAML boundaries were overfitted, shifting the age windows would cause irregular, random fluctuations in performance. Instead, we observed a consistent trend. The variance of association increased as the overlap proportion decreased, indicating greater fluctuation in performance when the age window shifted or widened (Fig. S3B). Furthermore, the overall association was positively correlated with the overlap proportion (*R* = 0.37, *P* = 4.36 × 10^–30^) (Fig. S3A). Collectively, these findings confirm that the AAML groups constitute the most explanatory and stable configurations, thereby reflecting robust model generalization rather than overfitting or artifacts.

### Identification of microbial candidates showing age-independent associations with skin quality

AAML identified microbial candidates hidden in standard age-group analyses. We found three microbes*—C*. *acnes*, *C. propinquum*, and *C. granulosum*—emerged as clear group-specific signals associated with skin quality, revealing relationships that were obscured in the whole-age analysis. AAML captures *C. acnes, C. propinquum*, and *C. granulosum* as a top candidate in each AAML-Young, AAML-Middle, and AAML-Old group, respectively (*z*-score: *C. acnes,* −3.744; *C. propinquum,* 4.345; and *C. granulosum,* 5.080) (Fig. 2B; Table S9). For further investigation, we assessed the association rank between skin quality and each AAML age group and the whole-age group (Tables S10 to S14; see Materials and Methods). *C. propinquum* ranked the highest for improved skin quality in the AAML-Middle group, whereas there were no signals in the whole-age model (AAML-Young: 23/25, None; AAML-Middle: 5/38, Improved; AAML-Old: 20/27, Deteriorated; Pearson: 44/58, Improved; Fisher: 20/40, Improved) (Fig. 2C). *C. acnes* was enriched among negative contributors in the AAML-Young group, whereas it shows the top candidates for improved skin quality in the whole-age model (AAML-Young: 8/25, Deteriorated; AAML-Middle: 23/38, Improved; AAML-Old: 19/27, Improved; Pearson: 25/58, Deteriorated; Fisher: 28/40, Improved; and Whole-age *z*-score: 3.822, Improved) (Fig. 2E). *C. granulosum* was the top positive contributor in the AAML-Old group, whereas it shows none of the signals for improved skin quality in the whole-age model (AAML-Young: 17/25, Deteriorated; AAML-Middle: 10/38, Deteriorated; AAML-Old: 6/27, Improved; Pearson: 45/58, Improved; and Fisher: 25/40, Deteriorated). (Fig. 2G).

AAML uncovers age-independent microbial associations that remain hidden when age effects are not considered (Tables S15 and S16). The presence of *C. propinquum* and *C. granulosum* showed no significant age bias across the cohort (*C. propinquum*: Old, *n* = 32 [0.51]; Young, *n* = 31 [0.49]; *C. granulosum*: Old, *n* = 101 [0.49]; and Young, *n* = 104 [0.51]) (Fig. S5B). Within their respective AAML age groups, however, both species were associated with a higher proportion of improved skin quality (*C. propinquum* in AAML-Middle: deteriorated, *n* = 7 [0.32]; improved, *n* = 15 [0.68]; *C. granulosum* in AAML-Old: deteriorated, *n* = 11 [0.38]; improved, *n* = 18 [0.62]) (Fig. S5D). In contrast, no significant difference in skin quality scores was observed between the presence and absence of *C. propinquum* or *C. granulosum* in whole-age analyses, indicating that microbial signals are only revealed in the AAML group (*P* = 0.78, *P* = 0.73) (Fig. S5G and H). In the case of *C. acnes,* it exhibited an age distribution bias toward younger individuals (Old, *n* = 21 [0.39]; Young, *n* = 33 [0.61]) and was associated with a higher proportion of deteriorated skin quality within the AAML-Young group (deteriorated, *n* = 8 [0.67]; improved, *n* = 4 [0.33]) (Fig. S5C). Consistently, *C. acnes* presence was associated with higher skin quality scores in whole-age analyses (*P* = 0.044) (Fig. S5F), illustrating how age-confounded effects can obscure microbial-skin relationships.

In contrast, in the whole-age analyses, we identified a potential risk of presenting microbe candidates associated with age-related confounding factors rather than with skin quality. *Streptococcus gordonii* was among the top 10 microbes with the highest absolute contribution scores in the deteriorated direction in the whole-age model (*z*-score = −2.559); however, it was not found in any AAML age group (Fig. S4A). Despite its apparent contribution in the whole-age analysis, *S. gordonii* does not represent a valid skin quality-related microbial candidate because its signal is driven by age-dependent bias rather than a direct association with skin condition. *S. gordonii* presence is biased toward older individuals and deteriorated skin quality. Among the samples, the presence of *S. gordonii* is biased toward the old group (Old, *n* = 82 [0.66]; Young, *n* = 42 [0.36]) as well as the deteriorated group in the whole-age group (deteriorated, *n* = 79 [0.66]; improved, *n* = 45 [0.36]) (Fig. S5E). These distributions indicate that the apparent association between *S. gordonii* and poor skin quality in whole-age analyses largely reflects age composition rather than a direct microbial effect.

### Investigation of the relationship between the microbial candidate and skin measurements

Our AAML model also accurately captures the well-known microbial associations, thereby enhancing its robustness and interpretability. *C. acnes* and *C. granulosum*, well-known contributors to skin quality, were identified with directions consistent with previous literature, validating the biological relevance of our results. *C. acnes* showed a negative association with moist, contributing to deteriorated moist compared to the whole-age group (Young: odds ratio [OR] 0.326, 95% CI [CI] 0.080–1.330, *P =* 9.8 × 10^–1^; Middle: OR 1.114, 95% CI 0.448–2.768, *P =* 0.5; Old: OR 1.0, 95% CI 0.134–7.460, *P =* 0.69, Whole: OR 0.688, 95% CI 0.134–7.460, *P =* 0.85) (Fig. 2F), consistent with its known inflammatory role in acne and reduced skin quality among younger individuals (28–30). Conversely, *C. granulosum* was significantly associated with improved skin oil in AAML-Old group (Young: OR 2.206, 95% CI 0.912–5.334, *P =* 6.1 × 10^–2^; Middle: OR 0.957, 95% CI 0.535–1.711, *P =* 0.96; Old: OR 3.263, 95% CI 1.252–8.499, *P =* 1.2 × 10^–2^; Whole: OR 2.028, 95% CI 1.188–3.46, *P =* 6.7 × 10^–3^), consistent with its role in lipid metabolism and sebum regulation (42, 43) (Fig. 2H). The recovery of these established associations reinforces the explanatory confidence and generalizability of AAML in capturing biologically interpretable microbial signals

Among the three microbial candidates, *C. propinquum* represents a newly identified microbe linked to improved skin tone in middle-aged individuals. We examined the specific association between skin-quality measurements and the presence of microbial candidates (Tables S17 to S19; see Materials and Methods). *C. propinquum* was significantly associated with skin tone improvement in AAML-Middle group, whereas the whole-age group does not show any association (Young: OR 0.299, 95% CI 0.057–1.580, *P* = 9.7 × 10^**–**2^; Middle: OR 2.383, 95% CI 0.972–5.844, *P =* 4.2 × 10^–2^; Old: OR 1.482, 95% CI 0.429–5.121, *P =* 0.38; Whole: OR 1.544, 95% CI 0.708–1.726, *P =* 0.18) (Fig. 2D; Fig. S6A).

We prioritized *C. propinquum* for downstream experimental validation over *C. acnes* and *C. granulosum* based on three key factors: novelty, phenotypic specificity, and mechanistic plausibility. (i) The beneficial impact of *C. propinquum* on skin quality represents a completely novel discovery. In contrast, the functional roles of the other top candidates are already well-established in the literature: *C. acnes* has been documented to have roles as an acne pathogen in youth, while *C. granulosum* is already documented for its association with improved skin oil and sebum regulation. (ii) *C. propinquum* emerged as a candidate specifically linked to improved skin tone in middle-aged individuals. Because middle age represents a critical window of accelerated skin changes (44–46)—unlike the stable states of youth and old age (35, 47–49)—it offers potential for microbiome-based cosmetic interventions. Collectively, the observed skin tone—improving the association of *C. propinquum* in this group—highlights its potential as a promising microbial candidate for cosmetic applications.

To ensure the statistical robustness of these findings against the zero-inflation inherent in sparse microbiome data (Fig. S1,3), our primary associations were evaluated categorically with Fisher’s exact test. However, to confirm that these signals persist when treating microbial abundance and skin measurements as continuous variables, we further validated our candidate associations using ALDEx2 (50) (see Materials and Methods).

This continuous statistical framework successfully mirrored our categorical results, preserving the group-specific rankings of our top candidates (Tables S20 to S23). Specifically, *C. propinquum* emerged as a top candidate for improved skin quality in the AAML-Middle group (AAML-Young: 9/18, Improved; AAML-Middle: 5/20, Improved; AAML-Old: 19/19, Improved; Whole: 12/18, Improved), while *C. propinquum* and *C. granulosum* remained the highest-ranked candidates for improved skin quality (AAML-Young: 5/18, Deteriorated; AAML-Middle: 6/20, Deteriorated; AAML-Old: 1/19, Improved; Whole: 6/18, Deteriorated) (Fig. S15C and E).

Furthermore, continuous differential abundance analysis exhibited consistent directional trends (effect sizes) for the specific phenotypic associations: *C. acnes* with moisture in the AAML-Young (Young: effect size −0.072, FDR *=* 0.99; Middle: Effect Size 0.012, FDR *=* 1; Old: NA, Whole: effect size −0.016, FDR *=* 1), *C. propinquum* with skin tone in the AAML-Middle (Young: effect size −0.069, FDR *=* 0.99; Middle: effect size 0.065, FDR *=* 0.95; Old: effect size 0.068, FDR *=* 0.99; Whole: effect size −0.033, FDR *=* 0.94), and *C. granulosum* with oil in the AAML-Old (Young: Effect Size 0.186, FDR *=* 0.12; Middle: effect size −0.024, FDR *=* 1; Old: effect size 0.347, FDR *=* 0.61; Whole: effect size 0.347, FDR *=* 0.61) (Fig. S15B, D, and F; Table S24). Demonstrating that these microbial signals reproduce the same biological inclinations across distinct mathematical frameworks (categorical presence/absence versus continuous abundance) provides directional support for our AAML-derived candidates.

### *In vitro* validation of *C. propinquum*-mediated effects on skin quality

**Fig 3:**
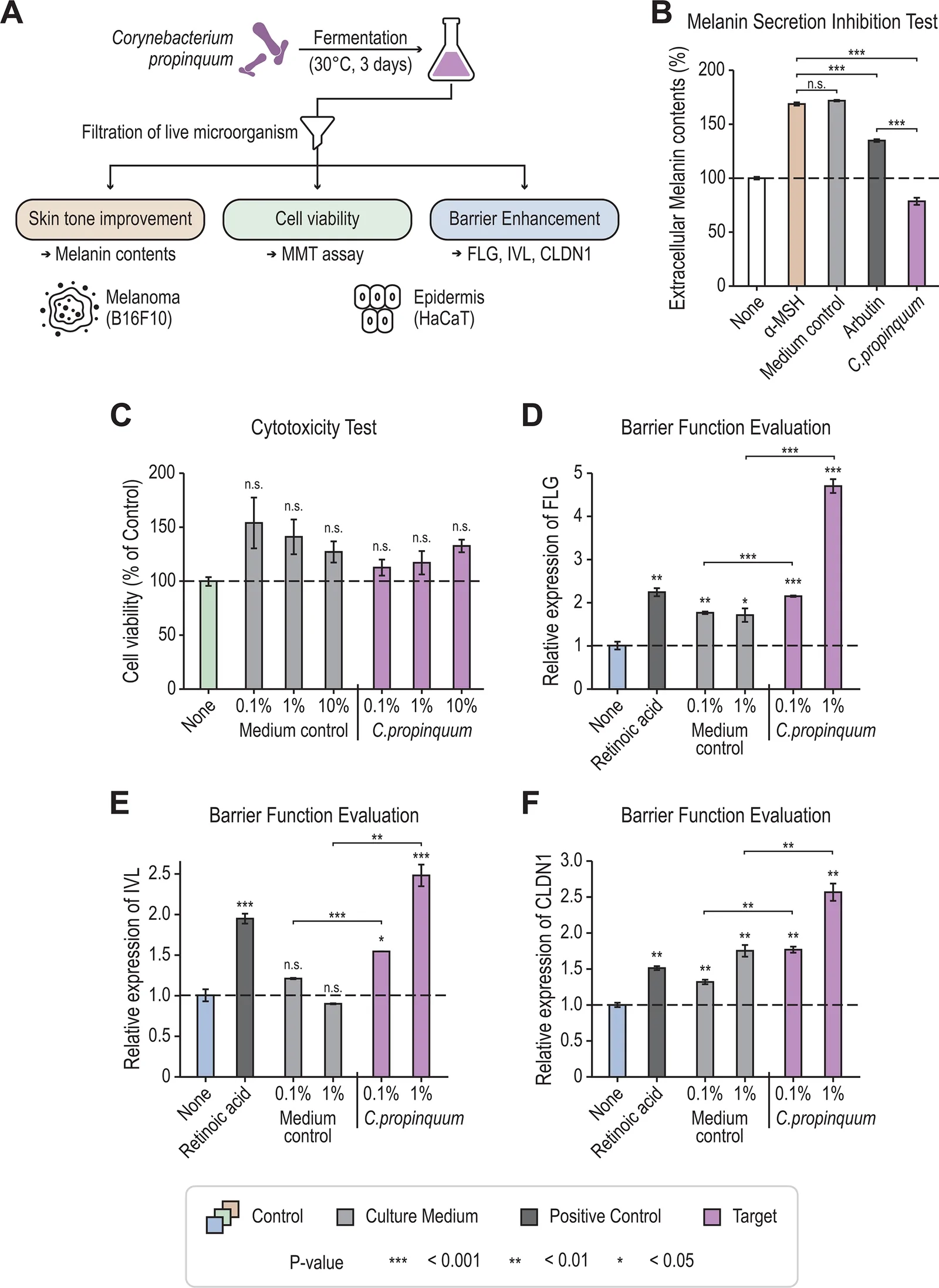
Biological effects of *C. propinquum* culture filtrate on skin-related cellular functions. (**A**) The experimental overview of *C. propinquum* culture filtrate effects on skin-related cellular function. (**B**) Bar graph of extracellular melanin contents of none, MSH-stimulated cells, MSH-stimulated cells with arbutin, MSH-stimulated cells with arbutin, and MSH-stimulated cells with *C. propinquum*. The statistical significance was tested using the *t*-test (*, *P* < 0.05; **, *P* < 0.001; ***, *P* < 0.001). (**C**) Bar graph of cell viability of none, TSB medium filtrate (control), and *C. propinquum* culture filtrate. The statistical significance was tested using the *t*-test (*, *P* < 0.05; **, *P* < 0.001; ***, *P* < 0.001). (**D–F**) Bar graph of the expression of epidermal barrier-related genes, such as FLG, IVL, and CLDN1, in none, retinoic acid, TSB medium filtrate (control), and *C. propinquum* culture filtrate. The statistical significance was tested using the *t*-test (*, *P* < 0.05; **, *P* < 0.001; ***, *P* < 0.001).

We experimentally confirmed that *C. propinquum* can enhance skin quality, including skin tone. We performed a series of *in vitro* functional assays targeting key dermatological outcomes relevant to cosmetic uses, such as improving skin tone, increasing cell viability, and strengthening the skin barrier (Fig. 3A; Table S25; see Materials and Methods). We assessed the whitening potential of *C. propinquum* to test its predicted association with improved skin tone directly. Treatment with 1% *C. propinquum* markedly suppressed melanin secretion compared with the MSH-induced control, reducing melanin content to 78.59% ± 3.29% versus 168.70% ± 1.47% (*P* = 1.32 × 10⁻¹⁵) (Fig. 3B). This inhibitory effect was stronger than that of the positive control arbutin (171.83% ± 0.66%; *P* = 4.28 × 10⁻¹¹), indicating potent anti-melanin secretion activity of *C. propinquum* culture filtrate through effective suppression of melanin secretion.

To further assess its cosmetic applicability, we evaluated cytotoxicity by measuring cell viability. Treatment with *C. propinquum* enhanced HaCaT cell viability in a concentration-dependent manner, increasing by approximately 12%, 17%, and 32% at 0.1%, 1%, and 10%, respectively, relative to untreated controls after 24 h. Even at the highest concentration (10%), viability did not fall below control levels, indicating the absence of cytotoxicity (Fig. 3C). These results confirm that *C. propinquum* is non-toxic to skin cells and can modestly promote keratinocyte viability.

In addition, we investigated barrier-enhancing effects, which are frequently co-improved with skin tone (51, 52), thereby extending the relevance of *C. propinquum* to broader cosmetic applications. We experimentally validated the barrier-enhancing effects of *C. propinquum* by assessing the expression of key epidermal barrier markers, including filaggrin (*FLG*), involucrin (*IVL*), and claudin-1 (*CLDN1*) (53–57). These proteins represent essential and complementary components of the epidermal barrier, contributing to keratin aggregation and maintenance of hydration (*FLG*), cornified envelope formation and mechanical stability (*IVL*), and tight junction-mediated regulation of paracellular permeability (*CLDN1*). Treatment with *C. propinquum* significantly upregulated FLG expression in a concentration-dependent manner, showing 2.15-fold ± 0.02-fold and 4.70-fold ± 0.16-fold increases at 0.1% and 1% (*P* = 2.4 × 10⁻⁴ and 3.6 × 10⁻⁵), comparable to or greater than retinoic acid (RA; 2.24-fold ± 0.09-fold; *P* = 3.3 × 10⁻³) (Fig. 3D). Similarly, IVL expression increased to 1.55-fold ± 0.00-fold and 2.48-fold ± 0.13-fold at 0.1% and 1% *C. propinquum* (*P* = 0.011 and 6.4 × 10⁻⁴), comparable to RA (1.95-fold ± 0.06-fold; *P* = 6.1 × 10⁻⁴) (Fig. 3E). Moreover, CLDN1, a major tight-junction protein, was upregulated by *C. propinquum* treatment, reaching 1.77-fold ± 0.04-fold (0.1%) and 2.57-fold ± 0.13-fold (1%) (*P* = 1.1 × 10⁻³ and 2.8 × 10⁻³), compared with 1.51-fold ± 0.03-fold in RA-treated cells (*P* = 1.5 × 10⁻³) (Fig. 3F). Overall, *C. propinquum* culture filtrate robustly enhances the expression of skin barrier-related genes, indicating its potential to reinforce epidermal integrity and barrier function.

Taken together, these results demonstrate that the AAML framework effectively identifies microbial candidates with biologically relevant effects on skin quality, supporting its utility for robust extraction of microbial candidates.

### Functional interpretation of *C. propinquum*-mediated skin tone improvement

**Fig 4:**
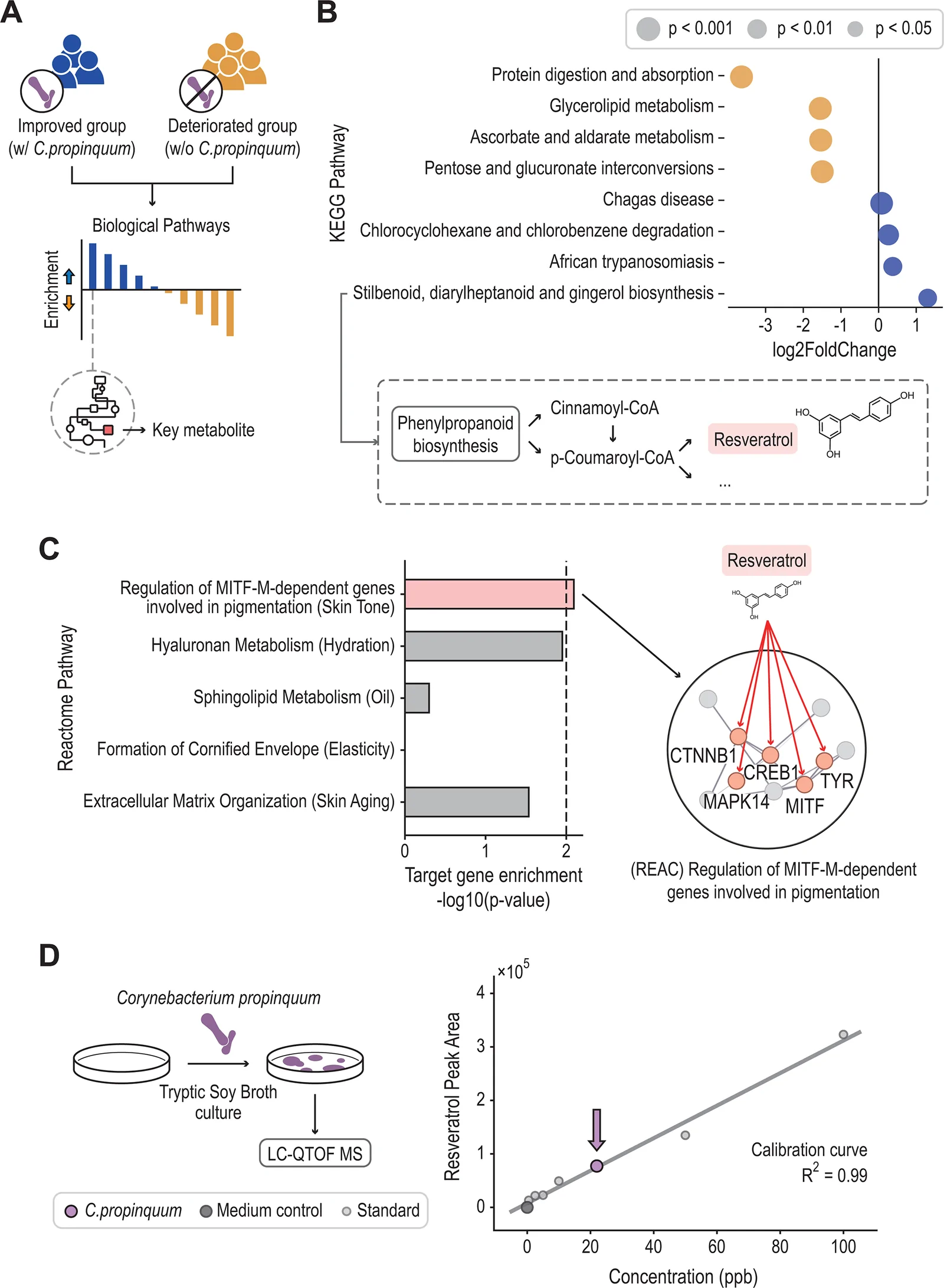
Gene functional mechanism of *C. propinquum* related to skin tone improvement. (**A**) The overview of gene functional analysis for *C. propinquum*. (**B**) The bubble plot for the enriched KEGG pathway in the *C. propinquum*-containing and improved skin quality group. The blue dot indicates the enriched pathway in the *C. propinquum* present and improved skin quality group (*n* = 17). Yellow dots indicate the enriched pathway in the *C. propinquum* absent and deteriorated skin quality group ( = 84). The bottom panel visualizes the partial chemical reaction involved in the “stilbenoid, diarylheptanoid, and gingerol biosynthesis” pathway. (**C**) The pathway enrichment test of the resveratrol target genes (*n* = 315) to the reactome pathway corresponding to the skin feature. The right panel indicates the resveratrol target genes in the skin tone-related pathway (regulation of MITF-M-dependent genes involved in pigmentation). (**D**) The left panel shows the overall process of the resveratrol-targeted LC-qTOF experiment from the *C. propinquum* culture. The right panel shows resveratrol concentration measurement of *C. propinquum* alongside the standard line. The purple dot indicates the resveratrol concentration in the *C. propinquum* culture. The gray-big dot indicates the TSB control concentration.

We interpreted the potential mechanism underlying skin-tone-improving effects of *C. propinquum*, focusing on resveratrol production. We conducted a differential expression analysis of KEGG orthologous groups (KOs) comparing samples containing *C. propinquum* and exhibiting enhanced skin quality with those lacking *C. propinquum* and demonstrating deteriorated skin quality. (Fig. 4A; see Materials and Methods). This analysis revealed significant enrichment of “stilbenoid, diarylheptanoid, and gingerol biosynthesis” pathways (ko00945) in the *C. propinquum*-positive, improved-skin group (log2 fold change = 1.303, *P* = 1.95 × 10^–5^) (Fig. 4B; Table S26).

Within this pathway, we prioritized resveratrol *in silico* based on metabolic parsimony (the shortest biosynthetic path) and phenotypic alignment (well-documented skin tone improvement function). Microbial systems optimize biosynthesis by minimizing the number of enzymatic steps (58–60). Therefore, we focused on two primary stilbenoids, pinosylvin and resveratrol, which are produced via a single enzymatic step (61, 62), whereas derivatives of other pathways require further downstream modifications. Between these two direct products, pinosylvin primarily reduces inflammation and lacks any documented effects on skin tone (63, 64). In contrast, resveratrol is a highly recognized stilbenoid known specifically for its skin tone improvement functions, aligning with our identified phenotype

Resveratrol is a well-characterized natural polyphenol widely incorporated into cosmetic formulations for its skin-enhancing properties, particularly its ability to improve skin tone by inhibiting melanogenesis and melanosome transport. Belonging to the stilbenoid subgroup of polyphenols, resveratrol has demonstrated multiple health benefits, including antioxidant and anti-inflammatory effects (65, 66). These activities contribute to its role in promoting skin health by reducing oxidative stress (67), protecting against UV-induced damage (68, 69), and facilitating skin regeneration (70, 71). In particular, resveratrol has been shown to enhance skin tone by suppressing melanosome transport and melanin synthesis (72–74). Mechanistically, resveratrol significantly targets the pigmentation pathway by regulating melanosome-associated genes, including *TYR* and *MITF* (regulation of *MITF*-M-dependent genes involved in pigmentation, *P* = 0.008) compared to moist (hyaluronan metabolism, *P* = 0.011), oil (sphingolipid metabolism, *P* = 0.382), elasticity (formation of cornified envelope, *P* = 1, and age-mediated pathway (49, 75)) (extracellular matrix organization, *P* = 0.029) (Fig. 4C; Fig. S1^1^A; Table S27). *TYR* encodes tyrosinase, the rate-limiting enzyme in melanin biosynthesis (76), while *MITF* is the master transcriptional regulator controlling pigmentation-related gene expression (77, 78). Downregulation of *TYR* and *MITF* is a well-established mechanism for improving skin tone. This prior knowledge aligns with our previous experimental data on the inhibition of skin melanosome transfer (Fig. 3). Consequently, these findings suggest that resveratrol could serve as the key metabolite linking *C. propinquum* to improved skin tone.

In contrast, whole-age analyses failed to capture metabolically significant microbial signals associated with skin quality, instead highlighting age-confounded associations. As mentioned earlier, *S. gordonii* is not a proper microbial candidate linked to skin quality due to confounded effects by age: biased to the old group as well as the deteriorated skin group (skin quality: *P* = 2.7 × 10⁻⁴, age: *P* = 3.9 × 10⁻⁷) (Fig. S7A). Consistent with this interpretation, samples lacking *S. gordonii* and exhibiting improved skin quality were enriched for pathways associated with age-mediated tissue remodeling and cellular structure, including extracellular matrix-receptor interaction (75) (log2 fold change = −2.137, *P* = 5.35 × 10⁻¹³), regulation of actin cytoskeleton (79, 80) (log2 fold change = −2.151, *P* = 5.35 × 10⁻¹³), focal adhesion (81, 82) (log2 fold change = −2.152, *P* = 5.35 × 10⁻¹³), and arrhythmogenic right ventricular cardiomyopathy (83) (log2 fold change = −2.152, *P* = 5.35 × 10⁻¹³) (Fig. S7B; Table S28). These pathways are well known to reflect aging-associated structural and mechanical changes rather than skin quality-specific mechanisms. This indicates that deteriorated signals of *S. gordonii* are induced by age-related effects, suggesting that whole-age analysis captures age-induced microbial signals rather than skin quality-related signals.

### Quantitative analysis of resveratrol produced by *C. propinquum*

We confirmed that *C. propinquum* produces resveratrol. To determine whether the strain synthesizes resveratrol, a quantitative analysis was performed using LC-QTOF mass spectrometry in negative ionization mode (Table S29; see Materials and Methods). The concentration of resveratrol in the Tryptic Soy Broth (TSB) culture of *C. propinquum* was about 22 ppb, while it was roughly 16 ppb in the Skin-Mimetic Broth (SMB) (Fig. 4D; Fig. S9D). Resveratrol was not detected in the uninoculated TSB medium used to culture *C. propinquum*, indicating that it is not derived from the culture medium itself but is produced by the strain’s metabolic activity.

We validated resveratrol derivative production in metabolite profiling via TSB and SMB (Tables S30 to S33; see Materials and Methods). We detected resveratrol derivatives, including resveratrol 4′-(6-galloylglucoside) from TSB cultures and trans-resveratrol 4′-O-glucuronide from SMB cultures (Fig. S8A and S9A). These metabolites are functionally relevant to resveratrol and the outcomes of the human metabolic pathway (84–86), showing additional confidence in resveratrol emission in *C. propinquum*.

We examined the effects of resveratrol on melanin secretion in B16F10 cells while assessing cytotoxicity to ensure that any observed effects were not due to decreased cell viability (Table S34). Resveratrol at 5 and 10 µM did not reduce cell viability (5 µM: 102.7% relative to control, *P* = 0.039; 10 µM: 101.4% relative to control, *P* = 0.304), whereas higher concentrations caused dose-dependent toxicity, lowering viability to 89.5% at 20 µM (*P* = 4.19 × 10⁻⁴) and 77.6% at 50 µM (*P* = 1.13 × 10⁻⁶) (Fig. S10B). Concentrations up to 10 µM were non-toxic and used for melanin assays. In α-MSH-stimulated B16F10 cells, extracellular melanin release rose to 109.9%. Resveratrol significantly reduced α-MSH-induced melanin secretion from the stimulated baseline of 109.9% down to 99.9% and 98.7% at 5 and 10 µM, respectively (5 µM: *P* = 2.06 × 10⁻¹⁰; 10 µM: *P* = 1.05 × 10⁻¹⁰, compared to the α-MSH treated group), while DMSO had no effect (*P* = 0.574) (Fig. S10A). These findings indicate that resveratrol inhibits melanin secretion at non-cytotoxic levels.

Together, these findings demonstrate that *C. propinquum*, identified by the AAML framework, can be mechanistically interpreted through a link between microbial metabolites, specific gene regulation, and skin phenotype. Our experimental validation confirmed that *C. propinquum* improves skin tone by producing resveratrol derivatives that inhibit melanin secretion by regulating *TYR* and *MITF*.

## DISCUSSION

In this study, we developed a framework to effectively identify the age-independent effects of skin microbiota on skin quality using AAML. By applying a machine learning approach that predicts skin quality from microbial composition through stratified age windows, we identified three different age groups with distinct skin microbial influences. The microbial candidates from AAML analysis showed notably strong associations with skin quality within each age group, whereas the overall-age analysis did not. Furthermore, this methodology facilitated the identification of dermatological characteristics that correlate with the presence of specific microbes. By diminishing age-related temporal heterogeneity, our approach is poised to uncover previously unrecognized microbial species that affect skin quality, thereby advancing future cosmetic research.

Our framework identified key species that confer benefits within age groups and linked these species to specific skin-improvement effects. We found that *C. propinquum*, which exerted a beneficial influence on the middle-aged AAML group, demonstrated a significant inhibitory effect on melanin secretion in human skin cell lines (Fig. 3B). Moreover, we verified its potential for development into cosmetic products by assessing safety and transcriptomic effects on epidermal barrier function. Given that middle-aged individuals are at a critical juncture with significant potential to improve skin quality, our findings are instrumental for identifying microbial candidates suitable for these populations. In contrast, young and elderly individuals exhibit distinguishable yet stable skin characteristics (35, 47–49); the middle-aged cohort undergoes accelerated changes, including collagen loss, structural modifications, and pigmentation issues (44–46). These findings underscore the potential of our approach to support the development of cosmetic products tailored for specific demographic groups based on skin microbiota.

The association between skin microbiota and the dermatological phenotype classified by AAML exemplifies the biological aspects of aging. Indeed, age is a key factor in the clinical field, and even guidelines often include it as a criterion due to its impact on biological processes (87–89). Furthermore, understanding the temporal dynamics is crucial for uncovering the effects of the microbiome on the host through host-microbiome interactions (90–94). Recent studies—especially those that emphasize the nonlinearity of biological aging, with rapid physiological and biological changes, including shifts in the skin microbiome (31–34)—indicate that biological aging does not always align precisely with chronological age despite their strong correlation. By optimizing age windows based on predictive performance, AAML identifies age ranges that likely correspond to shared biological aging states, thereby capturing the nonlinear temporal dynamics of microbiome-skin interactions and enabling more accurate prediction of skin quality from microbiome composition than whole-age analyses. Notably, the age boundaries where the effects of skin microbes differed, identified in our model (ages: 40 and 61), aligned with known molecular dysregulation points (ages: 44 and 60) documented in previous research (33). Consequently, this methodology facilitates the identification of microbial signals contingent on biological age within groups defined by chronological age.

Nevertheless, our research is limited in its capacity to definitively establish causal relationships between microbes and skin quality. Uncovering such causal relationships is vital for elucidating the biological mechanisms underlying dermatological conditions and for informing targeted therapeutic interventions. However, this remains challenging due to the complexity of host-microbiome interactions and confounding factors that impede accurate causal inference (95, 96). Nonetheless, we validated that the AAML approach generated biologically meaningful results that can facilitate further research in causal discovery. First, our methodology successfully replicated established bidirectional effects of skin microbiota across diverse age groups. The findings identified *C. acnes*, a representative microorganism that negatively affects skin quality exclusively in young individuals (25, 26, 28–30), as a contrasting contributor within the younger demographic and across other populations. Similarly, our approach provides insights into selecting known skin microbes, warranting further investigation of age-specific effects, such as *C. granulosum,* which exhibited opposite effects. Second, we conducted a gene-level functional analysis and proposed a mechanism by which *C. propinquum* influences skin tone enhancement (Fig. 3B). We experimentally validated that resveratrol, which targets genes involved in a biological pathway related to skin tone (70, 72–74), was produced by *C. propinquum* (Fig. 4C and D). However, given the significant influence of *C. propinquum* on skin tone, it should not yet be ruled out that other postbiotics, in conjunction with resveratrol, may have collectively contributed to the modulation of skin tone and hydration.

Our AAML approach can be applied to other cohort analyses where age may be a confounding factor. Given that our approach proved effective for the skin microbiome, which exhibits a significant correlation with age, this methodology may be beneficial in cohorts where a particular factor substantially influences the features of interest. To maximize accessibility and usability, we provide source code and a freely accessible web service (https://sbi.postech.ac.kr/skin_microbiome) that visualizes microbial associations with skin quality measurements across the AAML groups. We hope this resource will be useful for future research, including studies of human microbiome data.

## MATERIALS AND METHODS

### Sample collection

The facial rinse samples used in this study were derived from a previously published Korean cohort skin microbiome data set (39). All samples were collected from the entire faces of healthy Korean women without chronic skin diseases, such as atopic dermatitis and psoriasis. Detailed procedures for sample collection and clinical processing have been described in Mun et al. (39). Briefly, facial rinse sample collection was approved by the ethics committee of the H&BIO Corporation R&D Center Institutional Review Board (IRB Protocol Number: HBABN01-210217-HR-0181-01), and all clinical procedures were conducted in accordance with the guidelines and regulations of the Declaration of Helsinki. The data underlying this study are available in the original article and its Supplemental material.

### Data analysis of skin microbiome

The raw 16S rRNA sequence data utilized in this study were processed using the QIIME 2 pipeline. Briefly, following quality filtering and adapter removal, the Divisive Amplicon Denoising Algorithm 2 (DADA2) was implemented to correct sequencing errors and cluster the reads into Amplicon Sequence Variants (ASVs). To ensure high-confidence species-level taxonomic assignments, these ASVs were classified using a naive Bayes classifier trained on the V3-V4 hypervariable region of the SILVA 138 rRNA database, employing a strict 99% sequence identity threshold. Any ASVs matching Archaea, Eukaryota, mitochondria, or chloroplasts were explicitly filtered out to restrict the analysis to high-confidence bacterial taxa.

### Beta-diversity and PCA analysis

Beta-diversity was analyzed based on Bray-Curtis dissimilarities. To evaluate the associations and *R*² values between continuous skin measurements and microbial beta-diversity, the clinical variables were fitted onto the principal coordinates analysis (PCoA) ordination space using the envfit function in the vegan R package (ver. 2.7.1). To ensure rigorous statistical validity, the resulting *P*-values were subsequently adjusted for multiple comparisons using the Benjamini-Hochberg (BH) method to control the FDR. PCA analysis was conducted using the factoMine R package (ver. 2.11) and factoextra (ver.1.0.7). The following age-grouping criteria (20–44 years, 45–60 years, and 61–79 years) are based on previous research (6).

### Model training and evaluation

Microbiome data were obtained from the European Genome-Phenome Archive (EGA; accession EGAS00001007334). For skin quality classification, samples were divided into groups based on the OM_TE_score (see “Skin quality quantification” below).

As a preprocessing step for the machine learning models, microbial relative abundances were scaled to a uniform 0–1 range using MinMaxScaler in Python (ver. 3.11.5). It is a necessary procedure to ensure supervised learning consistency, prevent dominant species from disproportionately skewing model weights, and enable the direct comparability of feature coefficients (97). Model performance was assessed using the AUROC, with 5-fold cross-validation applied to prevent overfitting and enable statistical comparison.

Feature importance was estimated from regression coefficients: larger absolute weights indicated greater importance in the classification task (97). To calibrate feature importance across age groups, we calculated *z*-scored contribution scores as the mean across all five cross-validation folds. Positive values indicated predictive associations with the improved skin quality group, whereas negative values indicated predictive associations with the deteriorated skin quality group. These ranked coefficients were utilized to prioritize top microbial candidates for further evaluation.

### Model stability evaluation

The overlapped proportion score is calculated as follows:


$$Overlapped\;proportion\;score=\frac{overlapped\;years}{AAML\;age\;group\;length+target\;window\;length-overlapped\;years}$$

A highly overlapped proportion score indicates a high proportion of the age group length that overlaps with the AAML age group. The correlation is calculated using Pearson’s correlation, with a *P*-value. The variance is calculated within six bins of overlapped proportion score samples.

### Skin quality quantification

To characterize participants’ facial skin properties, a series of non-invasive measurements was conducted at baseline to assess epidermal hydration, transepidermal water loss (TEWL), sebum production, wrinkles, pores, skin color/tone, elasticity, and dermal density (39) (Table S35). During the conducting stage, several measures were taken to ensure accurate quantification of skin in localized areas. Due to strong correlations, skin tone (Color.Check.ITA) and elasticity (Elasticity.Cheek.R7) were merged into a “tone/elasticity” criterion, and oiliness (Oil.avg) and hydration (Hydration.avg and TEWL.avg) were merged into an “oil/moisture” criterion. The *z*-score (standard scalar, [SS]) is used as a relative measure.


$$SS\left(x\right)=\frac{20\;\times \left(x-x_{mean}\right)}{x_{std}}+100$$

The tone/elasticity criterion (TE.ss) and the oil/moisture criterion (OM.ss) are then used to calculate the skin quality score.


$$\begin{array}{rcl} {TE}_{SS} & = & SS\{SS(Color.ITA_{SS})\times SS(Elasticity.R7_{SS})\}\\ & = & SS\{SS({Color.Cheek.ITA}_{2})\times SS(Elasticity.Cheek.R7)\}\\ {OM}_{SS} & = & SS\{(Oi_{SS})\times SS(Moist_{SS})\}\\ & = & SS\left\{SS({Oil}_{avg})\times SS\left(\frac{{Hydration}_{avg}}{TEWL_{avg}}\right)\right\}\\ {Oil}_{avg} & = & \frac{Oil.Forehead+Oil.Nose+Oil.Cheek}{3},\\ {Hydration}_{avg} & = & \frac{{Hydration.Forehead}_{avg}+{Hydration.Cheek}_{avg}}{2},\\ TEWL_{avg} & = & \frac{TEWL.Forehead}{avg}+TEWL.{Cheek}_{avg} \end{array}$$

Two parameters, TE.ss and OM.ss, were combined into a single composite score via orthogonal projection using the scikit-spatial Python library (98). A reference line was defined in two-dimensional space using the lower and upper cutoff values. For each sample, the corresponding point was projected orthogonally onto this reference line, and the projected position along the line was used as the skin quality score, with higher scores indicating improved skin quality.

### Skin quality group classification

For whole-age classification tasks, the samples were divided into deteriorated and improved skin quality groups based on the skin quality score.

The method for classifying skin quality groups in the whole-age cohort is as follows:

- Linear regression between skin quality score and age.
- Improved skin quality group: top 40% distance between samples and the regression line.
- Deteriorated skin quality group: bottom 40% distance between samples and the regression line.
- Samples in the intermediate 20% (40%–60%) were excluded to establish distinct skin quality groups and strengthen the true microbiome signals, a common approach for intermediate continuous phenotypes (99) (Fig. S2C).

For the samples stratified into the AAML age group the following applied:

- Improved skin quality group: top 40% of OM_TE_scores.
- Deteriorated skin quality group: bottom 40% of OM_TE_scores.
- Samples in the intermediate 20% (40%–60%) were excluded to establish distinct skin quality groups and strengthen the true microbiome signals, a common approach for intermediate continuous phenotypes (99) (Fig. S3D).

These distinct grouping criteria were deliberately employed to isolate age-independent skin quality associations across different cohort models. For the AAML age-stratified groups, where age-confounding is inherently controlled by the narrow age window, a continuous binary rank-based method was utilized (100). Conversely, for the whole-age classification tasks, samples were divided using the residual distance from the linear regression between skin quality score and age. This age-adjustment via regression residuals is necessary to prevent age-biased predictions in unstratified cohorts (101, 102).

### Determination of the gray zone

The specific 20% “gray zone” was empirically determined by evaluating all samples in the data set. The samples in the intermediate 20% (40th–60th percentiles) of the total cohort were excluded across all model training processes. Because it represented the region with the minimum microbial compositional difference between samples above and below the median score (Fig. S1,2). The microbial compositional difference is calculated by adjusted Cohen’s distance (103). Consequently, isolating the top 40% and bottom 40% provided the optimal boundaries for defining the “Improved” and “Deteriorated” skin quality groups, ensuring robust and distinct biological signals.

### Age group selection and microbial candidate identification

To identify age windows with the strongest predictive performance, logistic regression models were trained on sliding age windows (10–30 years in size, advanced by 1 year per step) across the whole sample. This age boundary confinement strategy will minimize the serial dependence of age, reducing the risk for microbes common in younger skin, which are chosen as candidates for skin quality (Fig. 1C). For consistency, we restricted analyses to individuals aged 20–79 years (*n* = 595), ensuring balanced sample representation across age groups. The mean AUROC across 5-folds was calculated for each window.

We retained age groups that

1. showed significantly higher AUROC compared to models trained on the full age range (Fig. S3C) and
2. did not overlap with previously selected groups.

This process will eliminate the potential risk of arbitrary age group selection containing weak and age-independent associations between microbes and skin quality. This procedure yielded three non-overlapping age windows: AAML-Young (29–38 years), AAML-Middle (40–61 years), and AAML-Old (69–78 years).

Microbial candidates were selected from resident skin flora with the highest contribution scores in each group. This filtering step was applied to prioritize stable skin-resident microbes with established physiological relevance while minimizing the selection of transient or environmentally derived microbes (104). Resident taxa were defined as well-documented skin-associated genera, including *Cutibacterium* (30, 105, 106)*, Streptococcus* (106)*, Staphylococcus* (106, 107), *Corynebacterium* (108), and *Rothia* (109), which collectively represented approximately 75% of the total microbial abundance in the cohort (Fig. S14A).

### Association analysis between microbial presence and skin measurements

Associations between microbial presence and individual skin measurements (moist, oil, skin tone, and elasticity) were assessed using Fisher’s exact test to effectively account for the severe zero-inflation (absence) observed in the microbial abundance data (Fig. S1,3). Four categories were constructed: (i) improved vs deteriorated skin measurements and (ii) presence vs absence of each microbe.

The odds ratio (OR) was calculated to quantify the direction of association, with an OR > 1 indicating microbial enrichment in the improved skin group and an OR <1 indicating enrichment in the deteriorated group. Because our objective was to validate beneficial microbial candidates for skin quality, statistical significance was evaluated using a one-sided Fisher’s exact test with the direction specified as “greater.”

Associations between the skin quality score (OM_TE_score) and the microbiome were comprehensively assessed using Fisher’s exact test and ALDEx2 (50). For Fisher’s exact test, odds ratios (OR) were calibrated into a natural log scale to symmetrically quantify absolute differences between OR < 1 and OR > 1. The species rankings were subsequently determined based on absolute log-transformed odds ratios (Fisher’s exact test) and absolute effect sizes (ALDEx2).

### KEGG gene functionality analysis

To infer functional gene content, we used PICRUSt2 (ver. 2.5.2) to predict KEGG orthologous group (KO) abundances from 16S rRNA sequencing data. Pathway enrichment was assessed using the ggpicrust2 (110) package (ver. 2.3.2) in R (ver. 4.3.3).

Differential KO abundance between groups was computed as log2 fold change:


$$Log2\;fold\;change=log_{2}\left(\frac{\text{mean normalized abundance group 1 + 1}}{\text{mean normalized abundance group 2 + 1}}\right)$$

where a pseudocount of one was added to avoid division by zero. Pathways with adjusted *P* < 0.05 were considered significantly enriched. We used the ALDEx2 method for differential abundance analysis to calculate *P*-values.

The most significant pathway is identified using an adjusted *P*-value. If the adjusted *P*-value is identical, the pathway is designated with log2 fold change.

### Targeted enrichment analysis of resveratrol target genes across skin-feature-associated pathways

To assess whether resveratrol preferentially regulates skin feature-associated pathways, the targeted pathway enrichment analysis was performed using a hypergeometric test. A curated set of resveratrol target genes was obtained from the STITCH 5.0 database (111) and tested for enrichment against Reactome (112) pathways, with the union of all Reactome-annotated genes used as the background set. For each pathway, enrichment significance was calculated as


$$P=1-\sum_{i=0}^{k-1}\frac{\left(\frac{n}{i}\right)\left(\frac{M-n}{N-i}\right)}{\left(\frac{M}{N}\right)}$$

where k is the number of overlapping genes between the resveratrol target set and the pathway, n is the pathway size, N is the number of resveratrol target genes, and M is the background gene set size.

### Microbial isolation and culture of *C. propinquum*

The *C. propinquum* (Type strain: KACC 20833) was obtained from the Korean Agricultural Culture Collection (KACC; Wanju-gun, Jeonbuk-do, South Korea). A single colony of each bacterium was selected from the plate and grown overnight in a tryptic soy broth (TSB) or a SMB (113). The overnight culture was diluted 1:100 in fresh TSB or SMB medium (50 mL), transferred to a 250 mL Erlenmeyer flask, and incubated at 30°C on a shaker (160 rpm) in the dark for 48 h. After incubation, the TSB medium (control) and the *C. propinquum* culture at 1 × 10^8^ CFU/mL were centrifuged at 8,000 rpm for 10 min. They were filtered using a syringe filter (0.45-μm pore size; Minisart, Sartorius, Germany). All culture filtrates were prepared in triplicates.

### Cell line and cell culture (human keratinocyte HaCaT)

The HaCaT human keratinocyte cell line was obtained from the American Type Culture Collection (ATCC; Manassas, VA, USA) and maintained in Dulbecco’s modified Eagle’s medium (DMEM; HyClone, Logan, UT, USA) supplemented with 10% fetal bovine serum (FBS) and 1% antibiotic-antimycotic solution. The cells were cultured at 37°C in a humidified incubator with 5% CO_2_ and used for experiments at approximately 80% confluence. Before some treatments, HaCaT cells were pre-incubated with menthol (100 μM) in serum-free medium for 24 h to induce a controlled stress response.

### Cell viability assay

HaCaT cells were treated with various concentrations of *C. propinquum* culture filtrate (0.1%, 1%, and 10%), while B16F10 cells were treated with resveratrol (Sigma-Aldrich, MO, USA) at concentrations of 5, 10, 20, and 50 µM. Both treatments were performed in serum-free DMEM for 24 h. Cell viability was assessed by adding 20 μL of 3-(4,5-dimethyl-2-thiazolyl)-2,5-diphenyl-2H-tetrazolium bromide (MTT) solution (5,000 μg/mL) to each well, then incubating the plates for 4 h. Following incubation, the supernatant was removed, the formazan crystals were dissolved in 100 μL of dimethyl sulfoxide (DMSO), and cell viability was assessed by comparing absorbance at 540 nm with untreated controls.

### Barrier enhancement model and treatment

To evaluate the effects on barrier enhancement markers, HaCaT cells were treated with *C. propinquum* culture filtrate (0.1% or 1%) or 1 μM retinoic acid (RA) as a positive control in serum-free medium for 24 h. After treatment, the cells were harvested to analyze the expression of hydration-related genes, including filaggrin (FLG), involucrin (IVL), and claudin-1 (CLDN1).

To rigorously isolate the barrier-enhancing effects of microbial metabolites, the sterile microbial culture medium (TSB) was utilized as a strict negative control. Because TSB contains high concentrations of soy peptides and minerals (such as calcium) that are known to induce basal keratinocyte differentiation and upregulate barrier genes (e.g., FLG and CLDN1) independently (114, 115), the TSB control establishes a strictly elevated baseline. Consequently, any significant upregulation observed in the microbial filtrate groups represents a true, microbe-specific physiological effect that surpasses this basal nutritional stimulation.

### Cell line and cell culture (murine melanoma cells B16F10)

To analyze the anti-melanogenic effects of *C. propinquum* culture filtrate or resveratrol, a melanin content assay was performed. Murine melanoma cells B16F10 were obtained from ATCC. The cells were suspended in 2 mL of DMEM supplemented with 10% FBS, inoculated into six-well plates at a density of 1 × 10^6^ cells/well, and incubated at 37°C and 5% CO_2_ for 24 h until 40%–50% of the cells adhered to the bottom of the wells.

### Melanin content assay

B16F10 cells were treated with α-melanocyte-stimulating hormone (α-MSH, Sigma-Aldrich, MO, USA) at a concentration of 100 nM to induce melanogenesis. Subsequently, the *C. propinquum* culture filtrate (1%) or resveratrol (5, 10, and 20 µM) or DMSO (vehicle control, Sigma-Aldrich, MO, USA) was added to the cells and incubated for 3 days. Arbutin (100 ppm, Sigma-Aldrich, MO, USA) was used as a positive control. After 72 h of incubation, the culture medium’s optical density was detected at 490 nm using a microplate reader (Victor3, PerkinElmer, Waltham, MA, USA) to measure the amount of extracellular melanin secretion. Meanwhile, the remaining cells were harvested with 1 mL cold PBS and trypsin. Subsequently, the cells were counted using a hematocytometer (Paul Marienfeld, Lauda-Königshofen, Germany), and an equal number of cells (1  ×  10^6^  cells/mL) from each treatment group were collected. The cell suspensions were centrifuged at 1,000 rpm for 10 min to obtain cell pellets. The harvested cells were then dried at 60 °C for 1 h. A volume of 100 μL of 1 M NaOH solution (Sigma-Aldrich, MO, USA) containing 10% DMSO was added to each pellet to extract intracellular melanin. The absorbance of the resulting solution was measured at 490 nm using a microplate reader, and the melanin synthesis inhibition rate was subsequently determined.


$$Melanin\;contents\;\left(\%\right)=\frac{\text{average absorbance of each sample}}{\text{average absorbance of α-MSH treatment}}\times 100\;\left(\%\right)$$

### RNA extraction and real-time PCR

Total RNA was extracted from the cells using TRIzol reagent (Takara, Shiga, Japan) according to the manufacturer’s instructions. Residual RNA was further purified using the RNeasy Mini Kit (Qiagen, Hilden, Germany). Complementary DNA (cDNA) was synthesized from 1 μg of total RNA using reverse transcription premix (Elpis-Biotech, Daejeon, South Korea) under the following reaction conditions: 45°C for 45 min and 95°C for 5 min. Quantitative real-time PCR (qPCR) was performed using a StepOne Plus System Software (Applied Biosystems, Foster City, CA, USA) with SYBR Green PCR Master Mix (Applied Biosystems) according to the manufacturer’s instructions. The following gene-specific primers were used in the ABI 7300 system (Bioneer, Daejeon, South Korea) (Table S1).

The thermal cycling conditions were set as follows: initial activation at 50°C for 2 min and 95°C for 10 min, followed by 40 amplification cycles at 95°C for 10 s and 60°C for 1 min. The expression of β-actin was used as an internal control for normalization.

### LC-QTOF MS analysis for resveratrol quantification

Resveratrol quantification was performed using a liquid chromatography-quadrupole time-of-flight mass spectrometry (LC-QTOF MS) system (Tables S2 and S3). Lyophilized culture broth sample (0.1 g) was extracted with 1 mL of acetonitrile:water (4:6, vol/vol), followed by filtration through a 0.22-µm nylon membrane filter. Resveratrol standard (Sigma-Aldrich Production GmbH, Switzerland) solutions were prepared using the same solvent system at concentrations of 0.5, 2.5, 5, 10, 50, and 100 ppb for calibration curve construction. LC-QTOF MS analysis was conducted in negative electrospray ionization (ESI) mode, detecting the primary ion [M−H]^−^. Data acquisition parameters included retention time, *m*/*z*, exact mass, and peak area. Peak detection thresholds were set at an intensity greater than 500 counts. Metabolite identification was performed by matching exact mass and molecular formula against the Agilent Metabolite PCDL library (version 1.0, Agilent, CA, USA), with a minimum matching score of 70. Quantification was based on the calibration curve constructed from the resveratrol standard solutions, yielding a coefficient of determination (*R*²) of 0.99. The STORMS checklist can be accessed through this link: https://shorturl.at/JRa6i.

## Supplementary Material

Reviewer comments

## Data Availability

The data underlying this article are available in the article and in its supplemental material. The Korean cohort skin microbiome data are provided in the European Genome-phenome Archive (EGA; https://ega-archive.org/, EGA number: EGAS00001007334). The Python and R implementations of the AAML code and statistical analyses are available on GitHub at https://github.com/parksb0518/AAML.The main data underlying this article are also available on our companion website (https://sbi.postech.ac.kr/skin_microbiome). Additional data will be shared by the corresponding author upon reasonable request.

## SUPPLEMENTAL MATERIAL

Supplemental material msystems.00840-26-s0001.docx Tables S1-S3, description of Tables S4-S35, and supplemental figures.

Supplemental tables msystems.00840-26-s0002.xlsx Tables S4 to S35.

OPEN PEER REVIEW reviewer-comments.pdf An accounting of the reviewer comments and feedback.

ASM does not own the copyrights to Supplemental Material that may be linked to, or accessed through, an article. The authors have granted ASM a non-exclusive, world-wide license to publish the Supplemental Material files. Please contact the corresponding author directly for reuse.
