# Supplementary material for: Age-adjusted machine learning identifies facial skin microbes associated with skin quality among Korean women
Source: mSystems. 2026 Aug 20;11(9):e00840-26. doi: 10.1128/msystems.00840-26 (PMC13595949; doi:10.1128/msystems.00840-26)
Supplement: Supplementary file 1 [file reviewer-comments.pdf]

ASM | mSystems

Age-adjusted machine learning identifies facial skin microbes associated with skin quality among Korean women

Sangbeen Park, Hye-Been Kim, Hyunsoo Ahn, Geunyeong Lee, Woomin Song, Misun Kim, Eunjin Park, Byung Sun Yu, Miyang Han, Seyoung Mun, Dong-Geol Lee, Chun Ho Park, Seunghyun Kang, Hyung Woo Jo, and Sanguk Kim

Corresponding Author(s): Sanguk Kim, Pohang University of Science and Technology

Review Timeline:

Submission Date:	July 3, 2026
Editorial Decision:	July 16, 2026
Revision Received:	July 22, 2026
Accepted:	July 29, 2026

Editor: Shi Huang

Reviewer(s): The reviewers have opted to remain anonymous.

Transaction Report:

(Note: With the exception of the correction of typographical or spelling errors that could be a source of ambiguity, letters and reports are not edited. The original formatting of letters and referee reports may not be reflected in this compilation.)

DOI: https://doi.org/10.1128/msystems.00840-26

Re: mSystems00840-26 (**Age-adjusted machine learning identifies facial skin microbes associated with skin quality among Korean women**)

Dear Prof. Sanguk Kim:

Thank you for the privilege of reviewing your work. Below you will find my comments, instructions from the mSystems editorial office, and the reviewer comments.

The authors have addressed all reviewer comments comprehensively, including new analyses (FDR corrections, PCA re-analysis with non-redundant composites, ALDEx2 validation, empirical grey-zone determination) and honest correction of prior errors. The revision is responsive and the manuscript is substantially improved. I recommend acceptance without further external review, conditional on: (i) clarification of species-level taxonomic assignment from 16S data, (ii) consistent correction of "metagenomic" → "16S rRNA" throughout including figure legends, (iii) tempered framing of the ALDEx2 results, and (iv) correction of the residual typographical errors noted above. These items can be verified at the editorial/production stage.

Please return the manuscript within 60 days; if you cannot complete the modification within this time period, please contact me. If you do not wish to modify the manuscript and prefer to submit it to another journal, notify me immediately so that the manuscript may be formally withdrawn from consideration by mSystems.

Revision Guidelines

To submit your modified manuscript, log into the submission site at https://msystems.msubmit.net/cgi-bin/main.plex. Go to Author Tasks and click the appropriate manuscript title to begin. The information you entered when you first submitted the paper will be displayed; update this as necessary. Note the following requirements:

• Upload point-by-point responses to the issues raised by the reviewers in a file named "Response to Reviewers," NOT in your cover letter.
• Upload a compare copy of the manuscript (without figures) as a "Marked-Up Manuscript" file.
• Upload a clean .DOC/.DOCX version of the revised manuscript and remove the previous version.
• Each figure must be uploaded as a separate, editable, high-resolution file (TIFF or EPS preferred), and any multipanel figures must be assembled into one file.
• Any supplemental material intended for posting by ASM should be uploaded with their legends separate from the main manuscript. You can combine all supplemental material into one file (preferred) or split it into a maximum of 10 files with all associated legends included.

For complete guidelines on revision requirements, see our Submission and Review Process webpage. Submission of a paper that does not conform to guidelines may delay acceptance of your manuscript.

Data availability: ASM policy requires that data be available to the public upon online posting of the article, so please verify all links to sequence records, if present, and make sure that each number retrieves the full record of the data. If a new accession number is not linked or a link is broken, provide mSystems production staff with the correct URL for the record. If the accession numbers for new data are not publicly accessible before the expected online posting of the article, publication may be delayed; please contact production staff (mSystems@asmusa.org) immediately with the expected release date.

Publication Fees: For information on publication fees and which article types are subject to charges, visit our website. If your manuscript is accepted for publication and any fees apply, you will be contacted separately about payment during the production process; please follow the instructions in that e-mail. Arrangements for payment must be made before your article is published.

ASM Membership: Corresponding authors may join or renew ASM membership to obtain discounts on publication fees. Need to upgrade your membership level? Please contact Customer Service at Service@asmusa.org.

The ASM Journals program strives for constant improvement in our submission and publication process. Please tell us how we can improve your experience by taking this quick Author Survey.

Thank you for submitting your paper to mSystems.

Sincerely,
Shi Huang
Editor
mSystems

The authors have addressed all reviewer comments comprehensively, including new analyses (FDR corrections, PCA re-analysis with non-redundant composites, ALDEx2 validation, empirical grey-zone determination) and honest correction of prior errors. The revision is responsive and the manuscript is substantially improved. I recommend acceptance without further external review, conditional on:

(i) clarification of species-level taxonomic assignment from 16S data

We sincerely thank the Editor for requesting this methodological clarification. We agree that our original manuscript lacked sufficient detail regarding how high-confidence species-level assignments were derived from the 16S rRNA amplicon data.

To clarify, we utilized the rigorous bioinformatics pipeline established in the foundational cohort dataset paper (Mun et al., 2025, *Front. Cell. Infect. Microbiol.*). Specifically, to overcome the typical challenges of species-level resolution in 16S data, Amplicon Sequence Variants (ASVs) were classified using a Naïve Bayes classifier trained on the V3-V4 hypervariable region of the SILVA 138 rRNA database, employing a strict 99% sequence identity threshold.

To ensure complete methodological transparency for readers, we have added this detailed 16S rRNA processing pipeline directly to the Methods section.

[Line 524: **The raw 16S rRNA sequence data utilized in this study were processed using the QIIME 2 pipeline. Briefly, following quality filtering and adapter removal, the Divisive Amplicon Denoising Algorithm 2 (DADA2) was implemented to correct sequencing errors and cluster the reads into Amplicon Sequence Variants (ASVs). To ensure high-confidence species-level taxonomic assignments, these ASVs were classified using a Naïve Bayes classifier trained on the V3-V4 hypervariable region of the SILVA 138 rRNA database, employing a strict 99% sequence identity threshold. Any ASVs matching Archaea, Eukaryota, mitochondria, or chloroplasts were explicitly filtered out to restrict the analysis to high-confidence bacterial taxa.**]

(ii) consistent correction of "metagenomic" → "16S rRNA" throughout including figure legends

We sincerely thank the Editor for meticulous review. We apologize that a few instances of the term "metagenomic" inadvertently remained in the figure legends and supplementary table titles during our previous revision.

We have conducted a document-wide search of the entire manuscript, including all main text, figure legends, and supplementary materials, to ensure that every instance of "metagenomic" has been consistently corrected to "16S rRNA."

For transparency, we have highlighted a few of the key corrections below:

[Manuscript Line 1160: The profound effect of age on facial skin data and a framework for age-adjusted machine learning. (A) The curation of skin features and microbial abundance from **16S rRNA** sequencing data.]

We also revise the metagenome sequencing to 16s rRNA in supplementary materials.

[Supplementary Material Line 18: Supplementary Table 5. Microbiome abundance data from **16S rRNA** sequencing (Provide as a separate XLSX file)]

(iii) tempered framing of the ALDEx2 results

We sincerely thank the Editor for their constructive feedback regarding the framing of our new analyses. We agree that our previous phrasing (e.g., stating that the continuous framework "solidifies the reliability") was too strong, particularly given the lack of statistical significance (high FDR values) in the ALDEx2 results.

We have tempered the language in the Results section to clarify that the continuous framework provides qualitative, *directional support* for our categorical findings, rather than definitive statistical confirmation.

For transparency, we have revised the conclusion of this paragraph in the Results section as follows:

[Line 310: Demonstrating that these microbial signals reproduce the same biological inclinations across distinct mathematical frameworks (categorical presence/absence versus continuous abundance) **provide directional support for** our AAML-derived candidates.]

(iv) correction of the residual typographical errors noted above.

We thank the Editor for the opportunity to perfect the manuscript prior to publication. In addition to correcting the specific nomenclature errors regarding 16S rRNA, we have conducted a final, meticulous line-by-line proofreading of the entire manuscript, including all main text, figure legends, and supplementary materials. During this final review, we corrected any remaining minor typographical, grammatical, and formatting inconsistencies (e.g. double space elimination, fixed the missing parenthesis in the ALDEx2 results paragraph, and corrected the spelling of "Stilbenoid") to ensure the manuscript meets the high publication standards of *mSystems*.

Re: mSystems00840-26R1 (**Age-adjusted machine learning identifies facial skin microbes associated with skin quality among Korean women**)

Dear Prof. Sanguk Kim:

All comments have been addressed.

Your manuscript has been accepted, and I am forwarding it to the ASM production staff for publication. Your paper will first be checked to make sure all elements meet the technical requirements. ASM staff will contact you if anything needs to be revised before copyediting and production can begin. Otherwise, you will be notified when your proofs are ready to be viewed.

Data Availability: ASM policy requires that data be available to the public upon online posting of the article, so please verify all links to sequence records, if present, and make sure that each number retrieves the full record of the data. If a new accession number is not linked or a link is broken, provide production staff with the correct URL for the record. If the accession numbers for new data are not publicly accessible before the expected online posting of the article, publication may be delayed; please contact ASM production staff immediately with the expected release date.

Publication Fees: For information on publication fees and which article types have charges, please visit our website. We have partnered with Copyright Clearance Center (CCC) to collect author charges. If fees apply to your paper, you will receive a message from no-reply@copyright.com with further instructions. For questions related to paying charges through RightsLink, please contact CCC at ASM_Support@copyright.com or toll free at +1-877-622-5543. CCC makes every attempt to respond to all emails within 24 hours.

ASM Membership: Corresponding authors may join or renew ASM membership to obtain discounts on publication fees. Need to upgrade your membership level? Please contact Customer Service at Service@asmusa.org.

Embargo Policy: A press release may be issued as soon as the manuscript is posted on the mSystems Latest Articles webpage. The corresponding author will receive an email with the subject line "ASM Journals Author Services Notification" when the article is available online.

Cover Image Submissions: If you would like to submit a potential Cover Image, please email a file and a short legend to msystems@asmusa.org. Please note that we can only consider images that (i) the authors created or own and (ii) have not been previously published. By submitting, you agree that the image can be used under the same terms as the published article. Image File requirements: TIF/EPS, 7.5 inches wide by 8.25 inches tall (at least 2,250 pixels wide by 2,475 pixels tall), minimum 300 dpi resolution (600 dpi preferred), RGB, and no figure elements, e.g., arrows or panel labels. The legend should be a short description of the image, 1-2 sentences recommended. Please use the ASM Journals Cover Image Sizing Tool (https://journals.asm.org/pb-assets/html/asm-cover-sizing-tool.html) to ensure that your proposed cover image meets our size requirements.

Thank you for submitting your paper to mSystems.

Sincerely,
Shi Huang
Editor
mSystems
