# Supplementary material for: Age-adjusted machine learning identifies facial skin microbes associated with skin quality among Korean women
Source: mSystems. 2026 Aug 20;11(9):e00840-26. doi: 10.1128/msystems.00840-26 (PMC13595949; doi:10.1128/msystems.00840-26)
Supplement: Supplementary file 2 [file msystems.00840-26-s0001.docx]

**Supplementary Material**

**Age-adjusted machine learning identifies facial skin microbes associated with skin quality in Koreans**

Sangbeen Park^1†^, Hye-Been Kim^2†^, Hyunsoo Ahn^3†^, Geunyeong Lee^4^, Woomin Song^1^, Misun Kim^2^, Eunjin Park^2^, Byung Sun Yu^5^, Miyang Han^5^, Seyoung Mun^7,8,9^, Dong-Geol Lee^2^, Chun Ho Park^2^, Seunghyun Kang^6^*, HyungWoo Jo^2^*, and Sanguk Kim^1,3,4^*

**Contents**

**1. Supplementary Method**

**Supplementary Method 1. Detailed Methods of Biophysical Measurement for Skin Conditions**

**2. Supplementary Tables**

**Supplementary Table 1.** Primer sequences used for RT–qPCR analysis

**Supplementary Table 2.** Gradient Profile for the LC Method

**Supplementary Table 3.** Profile for the MS Method

**Supplementary Table 4.** Skin feature database (Provide as a separate XLSX file)

**Supplementary Table 5.** Microbiome abundance data from 16S rRNA sequencing (Provide as a separate XLSX file)

**Supplementary Table 6.** Beta-diversity of microbes (Provide as a separate XLSX file)

**Supplementary Table 7.** Skin quality score association single skin measurement (Provide as a separate XLSX file)

**Supplementary Table 8.** AAML performance of all age window (Provide as a separate XLSX file)

**Supplementary Table 9.** Microbial feature contribution (Provide as a separate XLSX file)

**Supplementary Table 10.** AAML-Young group skin-quality association rank (Provide as a separate XLSX file)

**Supplementary Table 11.** AAML-Middle group skin-quality association rank (Provide as a separate XLSX file)

**Supplementary Table 12.** AAML-Old group skin-quality association rank (Provide as a separate XLSX file)

**Supplementary Table 13.** Whole-age group skin quality association rank (fisher-test) (Provide as a separate XLSX file)

**Supplementary Table 14.** Whole-age group skin quality association rank (Pearson) (Provide as a separate XLSX file)

**Supplementary Table 15.** Skin quality association of microbial candidates by AAML age group and whole age (Provide as a separate XLSX file)

**Supplementary Table 16.** Age association of microbial candidates (Provide as a separate XLSX file)

**Supplementary Table 17.** Skin tone association of microbial candidates by AAML age group and whole age (Provide as a separate XLSX file)

**Supplementary Table 18.** Skin moist association of microbial candidates by AAML age group and whole age (Provide as a separate XLSX file)

**Supplementary Table 19.** Skin oil association of microbial candidates by AAML age group and whole age (Provide as a separate XLSX file)

**Supplementary Table 20.** Continuous microbiome abundance association with skin quality in AAML Young via ALDEx2 (Provide as a separate XLSX file)

**Supplementary Table 21.** Continuous microbiome abundance association with skin quality in AAML Middle via ALDEx2 (Provide as a separate XLSX file)

**Supplementary Table 22.** Continuous microbiome abundance association with skin quality in AAML Old via ALDEx2 (Provide as a separate XLSX file)

**Supplementary Table 23.** Continuous microbiome abundance association with skin quality in whole age via ALDEx2 (Provide as a separate XLSX file)

**Supplementary Table 24.** Skin measurement association of microbial candidates by AAML age group and whole age via ALDEx2 analysis (Provide as a separate XLSX file)

**Supplementary Table 25.** *In vitro* functional assay of *C. propinquum*-mediated effects on skin quality (Provide as a separate XLSX file)

**Supplementary Table 26.** KEGG functional enrichment test result of *C. propinquum* (Provide as a separate XLSX file)

**Supplementary Table 27.** Resveratrol target gene enrichment test result (Provide as a separate XLSX file)

**Supplementary Table 28.** KEGG functional enrichment test result of *S.gordonii* (Provide as a separate XLSX file)

**Supplementary Table 29.** Resveratrol targeted LC-qTOF results of *C. propinquum* (Provide as a separate XLSX file)

**Supplementary Table 30.** (negative, TSB) LC-qTOF screening results in *C. propinquum* (Provide as a separate XLSX file)

**Supplementary Table 31.** (positive, TSB) LC-qTOF screening results in *C. propinquum* (Provide as a separate XLSX file)

**Supplementary Table 32.** (negative, HSM) LC-qTOF screening results in *C. propinquum* (Provide as a separate XLSX file)

**Supplementary Table 33.** (positive, HSM) LC-qTOF screening results in *C. propinquum* (Provide as a separate XLSX file)

**Supplementary Table 34.** *In vitro* functional assay of resveratrol-mediated effects on skin quality (Provide as a separate XLSX file)

**Supplementary Table 35.** Details of skin measurements (Provide as a separate XLSX file)

**3. Supplementary Figures**

**Supplementary Figure 1.** Microbiome association with all skin measurements

**Supplementary Figure 2.** Detailed results of AAML (1)

**Supplementary Figure 3.** Detailed results of AAML (2)

**Supplementary Figure 4.** Skin feature database

**Supplementary Figure 5.** Detailed contribution score of AAML and whole age ML

**Supplementary Figure 6.** The association between skin features and microbial candidates

**Supplementary Figure 7.** Age-dependent microbe target investigation

**Supplementary Figure 8.** LC-qTOF mass spectroscopy results of *C. propinquum* in tryptic soy broth

**Supplementary Figure 9.** LC-qTOF mass spectroscopy results of *C. propinquum* in skin mimetic broth

**Supplementary Figure 10.** *In vitro* resveratrol melanin secretion inhibition and cytotoxicity

**Supplementary Figure 11.** Protein–protein interaction network of the MITF-mediated pigmentation pathway annotated with resveratrol target genes.

**Supplementary Figure 12.** Microbial dissimilarity across varying grey zone widths.

**Supplementary Figure 13.** Prevalence and zero-inflation of skin resident flora.

**Supplementary Figure 14.** Relative abundance of key skin resident flora.

**Supplementary Figure 15.** Continuous differential abundance analysis (ALDEx2) of the top microbial candidates with skin quality.

**Supplementary Methods**

**Supplementary Method 1. Detailed Methods of Biophysical Measurement for Skin Conditions**

**Measurement of skin hydration**

In this study, epidermal skin hydration of the facial area (forehead and cheek) was measured thrice using Corneometer® CM 825 (C+K, Germany) at baseline, and the average value was analyzed. The epidermis has the characteristic of having a high resistance to electricity. All electrical phenomena are caused by the movement of charges, and capacitance refers to the ability to store these moving charges.

In this device, an electric field is formed between the electrode plates of the probe, and the moisture content in the stratum corneum layer of the epidermis can be measured based on the capacitance generated at this time.

**Measurement of Transepidermal Water Loss (TEWL)**

The amount of transdermal water loss (TEWL) at each test site (forehead and cheek) was measured three times using the Tewameter® TM HEX (C+K, Germany) at baseline, and the average value of the stabilization section was analyzed. This device is an open chamber type with the top and bottom of the probe open, and moisture does not accumulate in the probe, allowing long-term measurement. The temperature and humidity sensor inside the probe measures the amount of water evaporation (g/h/m2) over time per unit area of the test site.

**Measurement of the amount of skin sebum**

The amount of skin sebum at the forehead, nose, left and right cheek sites was measured using the Sebumeter® SM815 (C+K, Germany) at baseline, and the average value of the stabilization section was analyzed. This device measures the amount of sebum per unit area (㎍/㎠) based on the principle of light reflection by contacting a translucent sebumeter cassette with the skin for about 30 seconds.

**Measurement of skin wrinkles (Lateral canthal lines and Nasolabial)**

3D image of the selected lateral canthal lines (eyes) and nasolabial (nose) was photographed using PRIMOSCR (Canfield, USA) at baseline. Parameters of the skin wrinkles (Ra value) were analyzed by PRIMOSCR Software.

**Measurement of the skin pore**

At baseline, the facial image for the cheek was taken in optical mode using Mark-Vu (Mark-Vu®;

PSI PLUS Co., Ltd., Suwon, Korea). The photographed optical image was analyzed by measuring the percentage (%) of the pore spot area for the analysis area of the cheek area using Image-Pro® 10 (Media Cybernetics, USA).

**Measurement of skin color and skin tone**

The skin color (lightness (L*), greenness (a*), and yellowness (b*)) and skin tone (ITA° value:

Individual Typology Angle) of the left or right cheek area was measured three times at baseline using Spectrophotometer® CM26dG (Minolta, Japan), and the average value was analyzed. The optical system of this device follows the colorimetric system of CIE L*a*b* by measuring the spectral reflectance and tristimulus values of the sample using the diffuse illumination 8° light receiving method equipped with the integrating sphere.

**Measurement of skin elasticity**

In this study, the skin of the left or right cheek area was measured using the Cutometer® MPA580 (C+K, Germany) at baseline. Skin elasticity was analyzed by the R2 parameter (Ua/Uf; gross elasticity or overall elasticity of the skin), R5 (Ur/Ue, the net elasticity), and R7, (Ur/Uf , the ratio of elastic recovery to the total deformation). This device measures and shows skin elasticity in graphs and values by absorbing the skin for 2 seconds 3 times in a row at a constant negative pressure of 450 mbar.

**Measurement of dermal density**

At baseline, the dermal skin density of the left or right cheek area was measured using the Ultrasound Probe of DermaLab® Series SkinLab Combo (Cortex Technology, Denmark). This device is based on an ultrasonic system intended to be used to visualize the layers of the skin.

**Supplementary Tables**

**Supplementary Table 1. Primer sequences used for RT–qPCR analysis.**

| Gene | Primer sequence (5′→3′) |
| --- | --- |
| β-actin | F: GGCCATCTCTTGCTCGAAGT; R: GACACCTTCAACACCCAGC |
| CLDN1 | F: CTGTCATTGGGGGTGCGATA; R: CTGACCAAATTCGTACCTGGC |
| FLG | F: TGAAGCCTATGACACCACTGA; R: TCCCCTACGCTTTCTTGTCCT |
| IVL | F: AATAACCACCCGCAGTGTCC; R: GCGGACCCGAAATAAGTGGA |

**Supplementary Table 2. Gradient Profile for the LC Method.**

| **LC Parameters** | | | |
| --- | --- | --- | --- |
| Instrument | Infinity 1290 II (Agilent, CA, USA) | | |
| Column | ZORBAX Eclipse Plus C18 RRHD 2.1×150 mm, 1.8 μm | | |
| Column Temp | 40 °C | | |
| Injection Volume | 5 μL | | |
| low Rate | 0.3 mL/min | | |
| Solvent | (A) 0.1% Formic acid in Water, (B) 0.1% Formic acid in ACN | | |
| Gradient | Time (min) | Solvent A (%) | Solvent B (%) |
|  | 0.0 | 95 | 5 |
|  | 0.5 | 95 | 5 |
|  | 30.0 | 5 | 95 |
|  | 35.0 | 2 | 98 |
|  | 40 | 2 | 98 |
|  | 40.1 | 95 | 5 |
|  | 45 | 95 | 5 |

**Supplementary Table 3. Profile for the MS Method.**

| **MS Parameters** | |
| --- | --- |
| Instrument | Agilent 6546 LC/Q-TOF |
| Ionization mode | Negative Dual AJS |
| Gas Temp | 210°C |
| Drying Gas | 6 L/min |
| Nebulizer pressure | 35 psi |
| Sheath Gas Temp | 380°C |
| Sheath Gas Flow | 11 L/min |
| Capillary Voltage | 3800V |
| Nozzle Voltage | 500V |
| Fragmentor | 200 V |
| Skimmer | 65 V |
| Acquisition Range | m/z 50 to 1,100 |
| MS acquisition Rate | 1 spectrum/sec |

**Supplementary Figures**

**Supplementary Figure 1.** **Microbiome association with all skin measurements.** The bar plot of beta-diversity plot of all skin measurements of **(A)** TEWL (transepidermal water loss), **(B)** Skin color (*L-value*: lightness, *a-value*: greenness, *b-value*: yellowness, and *ITA*: Individual Typology Angle), **(C)** Elasticity (*R2*: gross elasticity or overall elasticity, *R5*: the net elasticity, *R7*: the ratio of elastic recovery to the total deformation), **(D)** Hydration, **(E)** Skin oil, **(F)** density, wrinkle, and pore. The grey bar indicates the average value of each skin measurement. *1st*, *2nd*, *3rd*, and *avg* stand for the triplicate and average of the measure.

**Supplementary Figure 2. Detailed results of AAML (1). (A)** A PCA plot based on skin measurements of samples (*n* = 595) in the Korean women cohort. Samples are grouped and colored by age: 20–44 years (n = 238), 45–60 years (n = 171), and 61–79 years (*n* = 186). The ellipses represent the 80% confidence interval. The box plot shows differences in the distributions of the samples along the PC1 axis, and statistical significance was assessed using the Mann–Whitney U test (*, *P* < 0.05; **, *P* < 0.001; ***, *P* < 0.001; ns, not significant). **(B)** Bar plot of the sample size of each age group. 20s: 20-29, 30s: 30-39, 40s: 40-49, 50s: 50-59, 60s: 60-69, 70s: 70-79. **(C)** The scatter plot of samples with skin quality score and age. The black line is the regression line between skin quality score and age. Blue dot indicates improved skin quality samples. The yellow dot indicates deteriorated skin quality samples. **(D)** Volcano plot that shows correlation between skin quality score and each individual skin measurement.

**Supplementary Figure 3. Detailed results of AAML (2). (A)** Scatter plot of each age window with performance and overlap proportion with the AAML group. The color dot indicates the selected AAML group window. The red dot line indicates a linear regression line between overlap proportion and performance **(B)**. Bar plot of performance variance of age window within specific bins of overlap proportion **(C)**. Boxplot of the top 10 mean AUROC of the age window. The selected AAML groups are indicated in reds. Random indicates the performances using the samples with 10 ages randomly chosen with a specific random seed between 20 and 79. **(D)** The scatter plot shows the relationship between skin quality scores and samples categorized as improved or deteriorated within each AAML group. Sample numbers are indicated for each improved or deteriorated group within each age category. Blue indicates the improved skin quality group. Yellow indicates the deteriorated skin quality group.

**Supplementary Figure 4. Detailed contribution score of AAML and whole age ML (A)** heatmap containing contribution score within each age group and whole age group. The grey bar indicates the whole age ML feature score. The number in each heatmap tile indicates the rank of the absolute value of the contribution score.

**Supplementary Figure 5. Analysis of age-independent effects of skin quality score. (A)** An overview of the analysis investigating the age-independent skin effects of the target microbe. The proportions of young/old and improved/deteriorated skin quality samples will be measured among target-microbe-containing samples. **(B, C, D, E)** The proportion bar plot of young/old improved/deteriorated samples of each microbe. **(F, G, H)** The boxplot of skin quality score between target microbe-absent/present samples in the whole age group.

**Supplementary Figure 6. The association between skin features and microbial candidates.** The ratio bar plots of improved/deteriorated samples with microbial candidate presence. The bar plot is indicated within each age group. The corresponding microbe and skin feature is followed: **(A)** *C. propinquum* – Skin tone, **(B)** *C. granulosum* – Oil, **(C)** *C. acnes* – Moist, **(D)** *C. granulosum* – Moist

**Supplementary Figure 7. Age-dependent microbe target investigation. (A)** Bar plot of *S. gordonii* presence/absence between skin quality group and age group. **(B)** The bubble plot for enriched KEGG pathways in the *S. gordonii* contained and deteriorated skin quality group. A blue dot indicates the enriched pathway in the *S. gordonii* absent and improved skin quality group (*n* = 193). Yellow dots indicate the enriched pathway in *S. gordonii* present in the deteriorated skin quality group (*n* = 79).

**Supplementary Figure 8. LC-qTOF mass spectroscopy results of *C. propinquum* in tryptic soy broth*.* (A)** The abundance bar plot from LC-qTOF results emphasized with trans-resveratrol 4'-(6-galloylglucoside). **(B, C)** The abundance bar plot of all chemicals in positive and negative polarity from *C. propinquum*.

**Supplementary Figure 9. LC-qTOF mass spectroscopy results of *C. propinquum* in skin mimetic broth. (A)** The upper panel shows a skin mimetic broth, and the right panel shows the abundance bar plot from LC-qTOF results, highlighting trans-resveratrol 4'-O-glucuronide **(B, C)**. The abundance bar plot of all chemicals in positive and negative polarity from *C. propinquum* **(D)**. The scatter plot shows the resveratrol concentration measurements for *C. propinquum* alongside the standard line. The purple dot indicates the resveratrol concentration in the *C. propinquum* culture. The large grey dot indicates SMB control concentration.

**Supplementary Figure 10. *In vitro* resveratrol melanin secretion inhibition and cytotoxicity. (A)** Bar graph of extracellular melanin contents of none, MSH-stimulated cells, MSH-stimulated cells with Arbutin, MSH-stimulated cells with DMSO, and MSH-stimulated cells with resveratrol*.* Statistical significance was tested using the T-test (*, *P* < 0.05; **, *P* < 0.001; ***, *P* < 0.001). **(B)** Bar graph of cell viability for none, DMSO (control), and resveratrol. The statistical significance was tested using the T-test (*, *P* < 0.05; **, *P* < 0.001; ***, *P* < 0.001)

**Supplementary Figure 11. Protein–protein interaction network of the MITF-mediated pigmentation pathway annotated with resveratrol target genes. (A)** The STRING-derived protein–protein interaction network of genes involved in the Reactome pathway *Regulation of MITF-M–dependent genes involved in pigmentation*. Nodes highlighted in red indicate genes identified as resveratrol targets.

**Supplementary Figure 12. Microbial dissimilarity across varying grey zone widths.** The 20% grey zone (representing the exclusion of the intermediate 40th to 60th percentiles, leaving the top and bottom 40%) exhibits the minimum microbial dissimilarity, reflecting the highest level of biological ambiguity to be eliminated. The numbers represent the ratio of excluded samples to total samples. Avg. Hedges' g indicates the average adjusted Cohen's distance between samples within the defined grey zone.

**Supplementary Figure 13. Prevalence and zero-inflation of skin resident flora.** Bar plots illustrating the number of samples exhibiting true absence (zero-counts) versus presence for each targeted bacterial genus across the sample: (A) *Streptococcus* (B) *Staphylococcus* (C) *Rothia* (D) *Cutibacterium*, and (E) *Corynebacterium*

**Supplementary Figure 14. Relative abundance of key skin resident flora. (A)** The pie chart displays the compositional proportion of the designated resident skin genera, which collectively accounts for approximately 75% of the total microbial abundance in the cohort.

**Supplementary Figure 15. Continuous differential abundance analysis (ALDEx2) of the top microbial candidates with skin quality.** **(A, C, E)** Bar plots comparing the ALDEx2 association rank for skin quality scores between the age-adjusted analyses and the whole age samples statistical analysis. Blue upper arrow indicates the improved skin quality effects, and yellow downward arrow indicates the deteriorated skin quality effects **(B, D, F)** Bar plots displaying the ALDEx2 effect sizes for the associations between the individual microbial candidates and specific continuous skin measurements.
